# Identification of Cancer-Related Long Non-Coding RNAs Using XGBoost With High Accuracy

**DOI:** 10.3389/fgene.2019.00735

**Published:** 2019-08-09

**Authors:** Xuan Zhang, Tianjun Li, Jun Wang, Jing Li, Long Chen, Changning Liu

**Affiliations:** ^1^CAS Key Laboratory of Tropical Plant Resources and Sustainable Use, Xishuangbanna Tropical Botanical Garden, Chinese Academy of Sciences, Kunming, China; ^2^University of Chinese Academy of Sciences, Beijing, China; ^3^Department of Computer and Information Science, Faculty of Science and Technology, University of Macau, Macau, China; ^4^Institute of Medical Sciences, Xiangya Hospital, Central South University, Changsha, China

**Keywords:** cancer, long noncoding RNA, machine learning, Synthetic Minority Over-sampling Technique, XGBoost

## Abstract

In the past decade, hundreds of long noncoding RNAs (lncRNAs) have been identified as significant players in diverse types of cancer; however, the functions and mechanisms of most lncRNAs in cancer remain unclear. Several computational methods have been developed to detect associations between cancer and lncRNAs, yet those approaches have limitations in both sensitivity and specificity. With the goal of improving the prediction accuracy for associations of lncRNA with cancer, we upgraded our previously developed cancer-related lncRNA classifier, CRlncRC, to generate CRlncRC2. CRlncRC2 is an eXtreme Gradient Boosting (XGBoost) machine learning framework, including Synthetic Minority Over-sampling Technique (SMOTE)-based over-sampling, along with Laplacian Score-based feature selection. Ten-fold cross-validation showed that the AUC value of CRlncRC2 for identification of cancer-related lncRNAs is much higher than previously reported by CRlncRC and others. Compared with CRlncRC, the number of features used by CRlncRC2 dropped from 85 to 51. Finally, we identified 439 cancer-related lncRNA candidates using CRlncRC2. To evaluate the accuracy of the predictions, we first consulted the cancer-related long non-coding RNA database Lnc2Cancer v2.0 and relevant literature for supporting information, then conducted statistical analysis of somatic mutations, distance from cancer genes, and differential expression in tumor tissues, using various data sets. The results showed that our approach was highly reliable for identifying cancer-related lncRNA candidates. Notably, the highest ranked candidate, lncRNA AC074117.1, has not been reported previously; however, integrated multi-omics analyses demonstrate that it is the target of multiple cancer-related miRNAs and interacts with adjacent protein-coding genes, suggesting that it may act as a cancer-related competing endogenous RNA, which warrants further investigation. In conclusion, CRlncRC2 is an effective and accurate method for identification of cancer-related lncRNAs, and has potential to contribute to the functional annotation of lncRNAs and guide cancer therapy.

## Introduction

Cancer is a leading cause of death worldwide (Siegel et al., 2018) and it is established that cancers are caused by genetic and epigenetic changes (Kanwal and Gupta, 2010; You and Jones, 2012). Hence, high throughput technologies to characterize genes associated with cancer have applications with crucial implications for human health. Long non-coding RNAs (lncRNAs) account for the vast majority of non-coding RNAs longer than 200 nucleotides, and were previously considered “junk” RNA, due to their low coding potential; however, over recent decades, lncRNAs have been recognized as significant regulators of multiple major biological processes impacting development, differentiation, and metabolism (Bhan and Mandal, 2015). In cancer, lncRNAs act via multiple mechanisms, including regulation of chromatin topology in both cis and trans (chromatin remodeling, chromatin interactions), scaffolding of proteins and other RNAs, acting as protein and RNA decoys (competing endogenous RNA, ceRNA), regulating neighboring genes as natural antisense transcripts (NATs), and producing micropeptides (Aab et al., 2016; Ransohoff et al., 2018).

The aberrant expression of lncRNAs has been linked to typical cancer hallmarks, such as continuous proliferation, bypassing apoptosis, genomic instability, drug resistance, invasion, and metastasis (Renganathan and Felley-Bosco, 2017; Bhan et al., 2017; Balas and Johnson, 2018; Wang et al., 2019). For example, the lncRNA growth arrest-specific transcript 5 (*GAS5*), which is down-regulated in almost all tumor tissues, can suppress the tumorigenesis of cervical cancer by downregulating miR-196a and miR-205 (Yang et al., 2017), while *LncRNA-PVT1*, which is up-regulated in non-small cell lung cancer (NSCLC), can improve tumor invasion and metastasis (Yang et al., 2014). Further, Hox transcript antisense intergenic RNA (*HOTAIR*), which contributes to epigenetic regulation of genes, plays an important role in various cellular pathways by interacting with Polycomb Repressive Complex 2 (PRC2) (Mercer and Mattick, 2013). In addition, due to dynamic changes in their expression levels as cancer develops, some lncRNAs are regarded as potential biomarkers and therapeutic targets (Hanahan and Weinberg, 2011; Bhan et al., 2017). The most prominent example of such a biomarker is prostate cancer antigen 3 (*PCA3*), a lncRNA expressed at high levels in prostate cancer (De Kok et al., 2002; Yarmishyn and Kurochkin, 2015). The detection of *PCA3* in urine is a more specific marker for prostate cancer diagnosis than the commonly used factor, prostate specific antigen (PSA), and has been widely applied in the clinic (Hessels et al., 2003; Tinzl et al., 2004). Another example is lncRNA *TUC339*, which is highly enriched in extracellular vesicles secreted by hepatocellular carcinoma cells, where it regulates the growth and adhesion of tumor cells (Kogure et al., 2013). These features of lncRNA prompted us to search for efficient methods to predict functional lncRNAs in cancer, to facilitate deeper understanding of malignancies and the potential application of lncRNAs as targets for cancer therapies and diagnostics.

Systematic understanding of the contributions of lncRNAs to cancer is challenging, partly due to the unpredictability of lncRNA functional elements, as well as their relatively low conservation, low expression levels, and diverse functional mechanisms. The functions of a single lncRNA, or several lncRNAs, can be determined using experimental methods; however, this approach is time consuming and costly. The successful implementation of machine learning systems for the study of genomics, proteomics, systems biology, and evolution, has been a great inspiration to the field of life sciences more generally (Larranaga et al., 2006). Using machine learning algorithms, we can determine the high dimensional characteristics of functional lncRNAs from an informatics perspective. To successfully apply machine learning to the identification of functional lncRNAs in cancer genomics, it is fundamental to first identify positive and negative sets. For this purpose, there are a number of repositories from which cancer-related lncRNAs can be conveniently obtained, including Lnc2Cancer v2.0, a manually curated database that provides comprehensive experimentally supported associations between lncRNAs and human cancer (Gao et al., 2019), and CRlncRNA, another manually curated database that uses stricter criteria to retain only data related to cancer hallmarks that have been experimentally confirmed (Wang et al., 2018). These databases can be exploited to develop machine learning models to predict and rank cancer-related lncRNAs. There has been relatively little research that has attempted to use machine learning methods to predict functional lncRNAs in cancer. For example, Zhao et al. (2015) presented the first naïve Bayes based machine learning method, and identified 707 cancer-related lncRNA candidates. In our previous work, we used a Random Forest based algorithm, CRlncRC, to classify cancer-related lncRNAs and other lncRNAs, through integration of 85 features (Zhang et al., 2018); however, compared with the computational prediction work reported for cancer-related protein-coding genes, the identification of cancer-related lncRNAs remains preliminary. The sensitivity and specificity of methods to predict cancer-related lncRNAs require further improvement.

In this study, we developed a new cancer-related lncRNA classifier, CRlncRC2. Compared with CRlncRC, CRlncRC2 uses the Laplacian score feature selection method to reduce training time and prevent over-fitting. In addition, unlike the naïve under-sampling method adopted by CRlncRC, we address the data imbalance problem, which is caused by the relatively small size of available positive sets of cancer-related lncRNAs, using the Synthetic Minority Over-sampling Technique (SMOTE) method, to balance imbalanced data, while aiming to retain all important information. Moreover, CRlncRC2 uses a more powerful machine learning model, extreme gradient boosting machine (XGBoost), to improve its predictive performance. Ten-fold cross-validation showed that the area under the receiver operating characteristic curve (AUC or area under ROC curve) score of CRlncRC2 is much higher than those of CRlncRC (0.86 vs. 0.82) and the method developed by Zhao et al. (0.90 vs. 0.79). Finally, 439 possible cancer-related lncRNAs were identified using CRlncRC2, of which 5 in the top 20 were confirmed using the Lnc2Cancer v2.0 database. Further, statistical analyses show that the identified lncRNAs are closer to cancer protein genes, carry more mutations, and are more likely to be differentially expressed in tumor tissues than negative lncRNAs. In addition, survival analysis revealed a significant difference in overall survival between the low and high expression groups of the top 10 predictions. In particular, one lncRNA, AC074117.1 (ENSG00000234072), which was the top ranked of our predictions and has not been reported in the literature, is suggested as being highly likely to be associated with cancer in the lncRNA-related ceRNA network. In conclusion, CRlncRC2 exhibited good performance in both cross-validation and prediction evaluation. We believe our framework will be a useful tool for study of lncRNA–cancer associations.

## Materials and Methods

**Figure 1:**
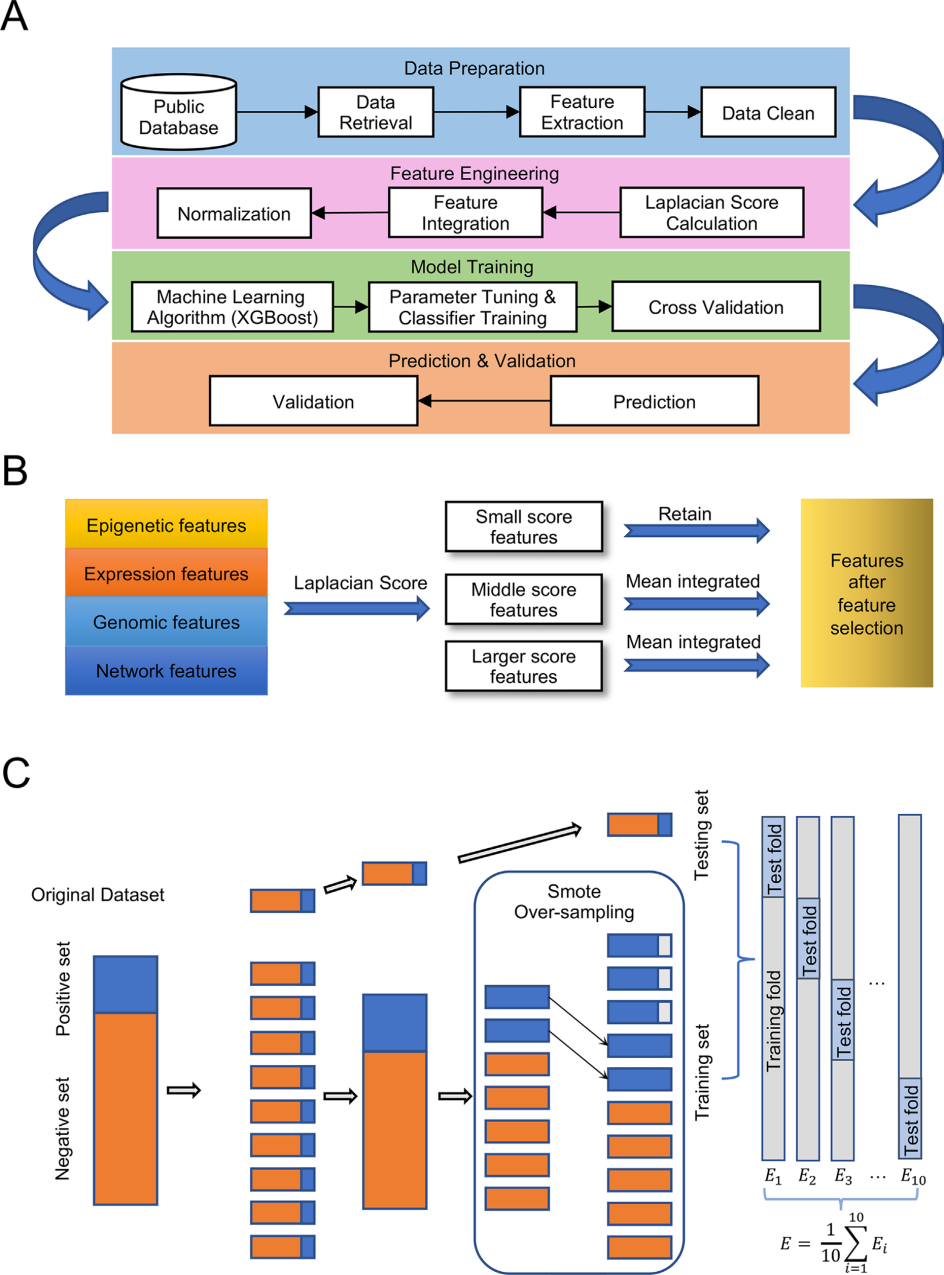
Pipeline for the experiment. **(A)** Designment of experimental workflow. **(B)** Details of feature selection. **(C)** Details of 10-fold cross validation with over-sampling.

Our experiment followed the pipeline illustrated in **Figure 1A**, which consisted of four main steps: Data preparation, Feature engineering, Model training, and Prediction and validation. The detailed processes of feature selection and cross-validation are presented in **Figures 1B, C**.

### Data Preparation

Cancer-related lncRNAs (positive set) and cancer unrelated lncRNAs (negative set) were downloaded from CRlncRC (https://github.com/xuanblo/CRlncRC). The criteria for cancer-related lncRNA collection include either differentially expressed in cancer (as verified by Real-Time qRT-PCR), co-occurred with a significant relevant clinicopathological parameter (e.g., tumor differentiation, clinical stage, and survival time), or proven by functional experiments (e.g., colony formation assay, matrigel invasiveness assay, xenograft mouse model, and metastasis nude mouse model). As the category of cancer unrelated lncRNA is difficult to define, and for consistency with other classifiers, we located a large number of single-nucleotide polymorphisms (SNPs) associated with phenotypes derived from the NHGRI-EBI GWAS Catalog (Welter et al., 2014) in the sequences of lncRNAs, and only those lncRNAs which had no phenotype-related SNPs detected within its 10 kb up/down stream were selected as cancer non-related lncRNAs. Finally, we identified 158 positive lncRNAs (**Data Sheet 1**) and 4,533 negative lncRNAs (**Data Sheet 2**).

We downloaded lncRNA feature data from CRlncRC; CRlncRC retrieves 85 features and groups them into four categories: genomic features, expression features, epigenetic features, and network features. Feature category, name, source database, and description information are detailed in **Data Sheet 3**.

### Feature Engineering

Features play an essential role in classification, and appropriate features can improve classification performance significantly. In cancer genomic research, the currently known cancer-related lncRNA (positive) set are only available because they were identified by humans. It is possible that some samples in the negative set may be considered to belong to the positive set in the future. Hence, we employed Laplacian scoring (He et al., 2005), which is designed to select features without labels, as a criterion to evaluate the correlations of each feature. The basic idea of Laplacian score is to evaluate the features according to their locality preserving power, which is from the Laplacian Eigenmaps (Chung, 1997) and Locality Preserving Projection (He and Niyogi, 2003).

**Figure 2:**
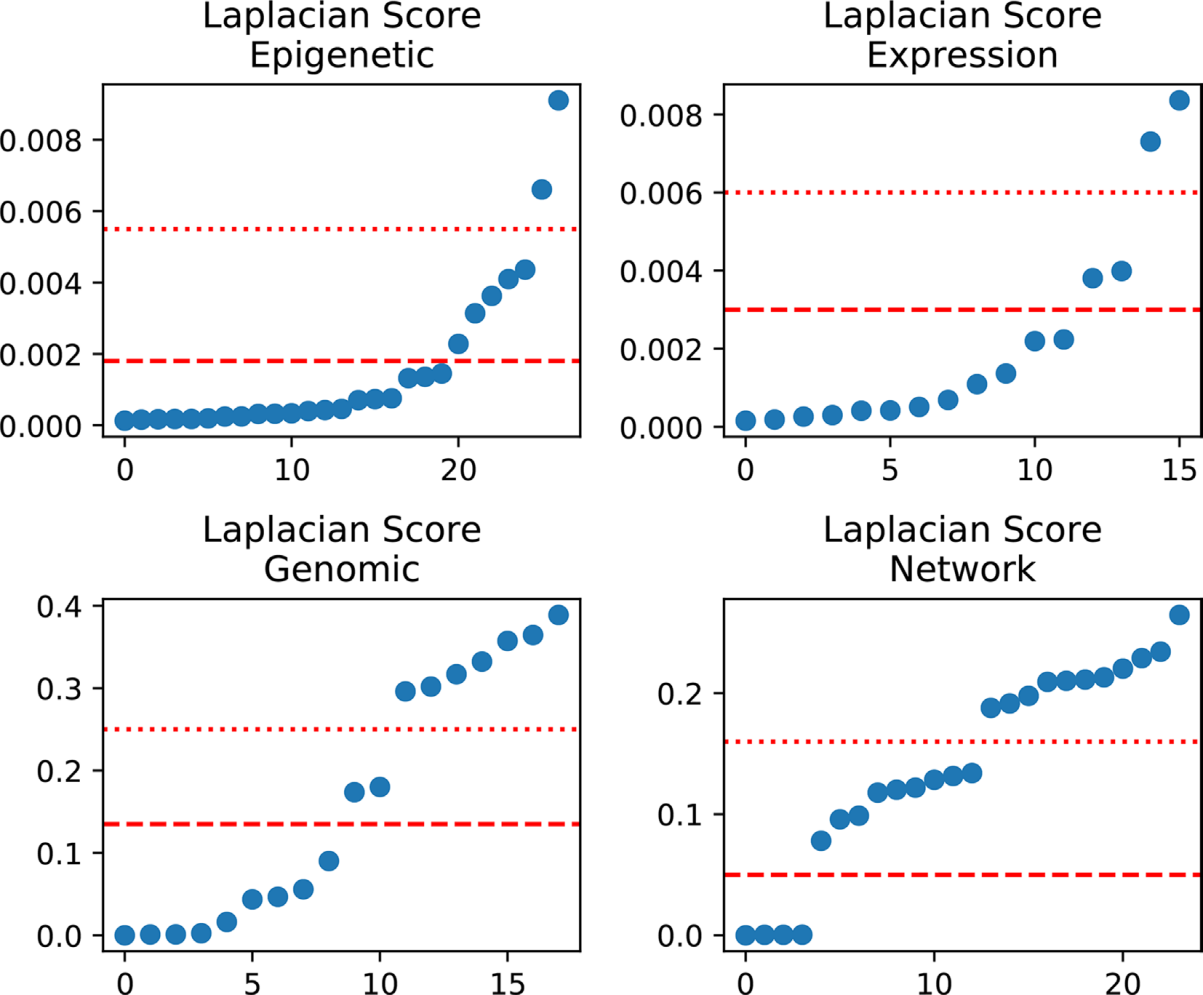
Laplacian score distribution. Right, sorted scores (asc). Red dotted line and dashed line, assumed thresholds.

In detail, we applied the scikit-feature (Li et al., 2017) to calculate Laplacian scores; the parameters for the affinity matrix used for the calculation are as follows: metric = euclidean, neighbor mode = knn, and k = 5. Calculated scores range from 0 to 1, with smaller values indicating more important features. The distribution of calculated Laplacian scores is presented in **Figure 2** and clearly shows that there are large margins in each category of features. In this case, we can determine the diﬀerence between the sorted Laplacian scores (asc) and use the first two differential values to set a threshold. Specifically, we set the margins in “Epigenetic” to the second and third largest diﬀerential values, because these appeared to be the inflection points. Hence, the features were split into three parts, and the features located in the lower part (i.e., those with scores indicating that the features are more important) retained immediately. Nevertheless it is not advisable to simply remove those features located in the other parts, as these also contain some information. Therefore, we merged the features according to the mean in each part and retained the merged features to preserve the information. For example, the middle scoring part of “Expression” contains two features, and we removed these two features, while retaining their mean value. The mean-merged feature obtained from the high scoring parts were also retained. Finally, generated training and validation sets by concatenating the processed category features. Changes in the feature number in each category are summarized in **Table 1**. After feature selection, we obtained 51 features, eight of which are synthetic. A “Bigtable”, containing 11194 lncRNAs from CRlncRC, with 85 features, is included in **Data Sheet 4**.

**Table 1:** Changes in feature number for each type of feature.

Epigenetic			Expression			Genomic			Network		
Feature number before feature selection											
LP*	MP*	UP*	LP*	MP*	UP*	LP*	MP*	UP*	LP*	MP*	UP*
20	5	2	12	2	2	9	2	7	4	9	11
Feature number after feature selection											
22			14			11			6		

*LP, lower part of Laplacian Score; MP, middle part of Laplacian Score; UP, higher part of Laplacian Score.

### Model Training

The machine learning method, XGBoost (Chen and Guestrin, 2016), was tuned to search for an optimal prediction solution. XGBoost is a type of gradient boosting decision tree method; its objective function is defined in equation (1).

(equation 1) $$ℒ(\phi )=\sum_{i=1}^{n}\mathrm{loss}(y_{i}{\hat{y}}_{i})+\sum_{k=1}^{K}\Omega (f_{k})\text{,}$$

where loss is the training loss, Ω(*f*) is the complexity of the tree, and K is the number of trees in the model. This model can be optimized by minimizing this objective function. To this end, an additive training method was employed for training loss, and prediction at the additive *t* *^th^* training round could be quickly optimized using Taylor expansion. The greedy algorithm [31] was used to determine optimal tree complexity.

In our study, we used the dmlc XGBoost library (https://xgboost.ai/) for implementation of the XGBoost model. To tune the hyper-parameters, we first adopted Bayesian optimization to search for potential hyper-parameters and then manually fine-tuned those hyper-parameters to improve the performance of the model. The hyper-parameters for XGBoost primarily control the growth and the robustness of the model:

- Growth: n estimators, max depth, and learning rate
- Robustness: colsample bytree, subsample, and gamma

In addition, as our sample was unbalanced (the ratio of the minority positive class versus majority negative class was approximately 1/30), we adopted SMOTE (Nakamura et al., 2013) to re-sample our training set by Bayesian optimization, which reduces the impact of data imbalance. The final tuning result for this model is n estimator = 546, max depth = 10, learning rate = 0.01, colsample bytree = 0.7, subsample = 0.826, and gamma = 0.036.

Ten-fold cross validation was adopted to evaluate the model trained by parameters obtained using Bayesian optimization. The algorithm stratified shuffles the total samples into 10 folds, and begins an iteration: each time 9 folds are initially over-sampled, and then assigned for training. The single remaining fold is adopted as the pair for validation. Subsequently, the over-sampled training set was used to fit the model, while the validation set was utilized to evaluate the model’s performance. Note that the validation set in each iteration is not re-sampled and does not include any data used for training. Further, the models trained on each iteration are independent of one another. To rigorously evaluate the performance of our model, we measured the AUC scores using the abovementioned 10-fold cross-validation (**Figure 1C**).

Further, to rigorously evaluate the model’s performance, we measured the recall, precision, and F1 score, using the 10-fold cross-validation process described above.

The recall is the ratio of correctly predicted positive observations to all observations in a specific class, and was calculated using equation 3:

(equation 2) $$\mathrm{Recall}=\frac{\mathrm{TP}+\mathrm{FN}}{\mathrm{TP}}\text{,}$$

The precision is the ratio of correctly predicted positive observations to total predicted positive observations, and was calculated using equation 4:

(equation 3) $$\mathrm{Precision}=\frac{\mathrm{TP}+\mathrm{FP}}{\mathrm{TP}}\text{,}$$

The F1 Score is the weighted average of Precision and Recall, and was calculated using equation 5:

(equation 4) $$F1\;\mathrm{score}=2*\frac{\left(\mathrm{Recall}*\mathrm{Precision}\right)}{\left(\mathrm{Recall}+\mathrm{Precision}\right)}\text{,}$$

### Prediction and Evaluation

To predict novel cancer-related lncRNAs, we used our pre-trained model to predict 7,253 unknown lncRNAs from TANRIC [33]. To evaluate the accuracy of our model, we used various methods to test the reliability of our predictions. First, predictions were searched against the Lnc2Cancer v2.0 database. Next, the Kolmogorov-Smirnov test was used to examine whether there were significant differences among the different sets (positive, negative, and predictive) in the distance to cancer protein-coding genes, mutation numbers, and numbers of samples differentially expressed between tumor and normal tissues. Mutation data and cancer protein-coding gene sets were download from COSMIC [34]. Tumor and normal tissue expression profiles were downloaded from TANRIC. Further, survival analysis for the top 10 predictions was conducted using TANRIC.

## Results

### Data Collection

We collected 158 highly trusted cancer-related lncRNAs from CRlncRC as our positive data set. All have been reported in the literature with the support of strict experimental validation and are involved with cancer hallmarks. lncRNAs (n = 4,553) in CRlncRC without phenotype-related SNPs within 10 kb up- or down-stream were used as our negative data set. In CRlncRC, we collected 85 features that could potentially facilitate the recognition of cancer-related lncRNAs and grouped them into four different categories (see **Data Sheet 3** for details): Genomic features (such as GC content and sequence conservation score), Expression features (the expression profiles of lncRNAs in 16 different tissue types), Epigenetic features (different types of epigenetic signals in different types of cell lines), and Network features (the interactions between lncRNAs and cancer-related protein-coding genes and miRNAs). After feature selection using Laplacian scores, we reduced the feature number from 85 to 51. Cumulative curves were plotted and showed that the distribution of the feature values between the positive and negative sets was significantly different (Kolmogorov-Smirnov test, p-value < 0.05) (**Data Sheet 5**). The number in each feature category before and after feature selection is shown in **Table 1**.

### Performance Evaluation

**Figure 3:**
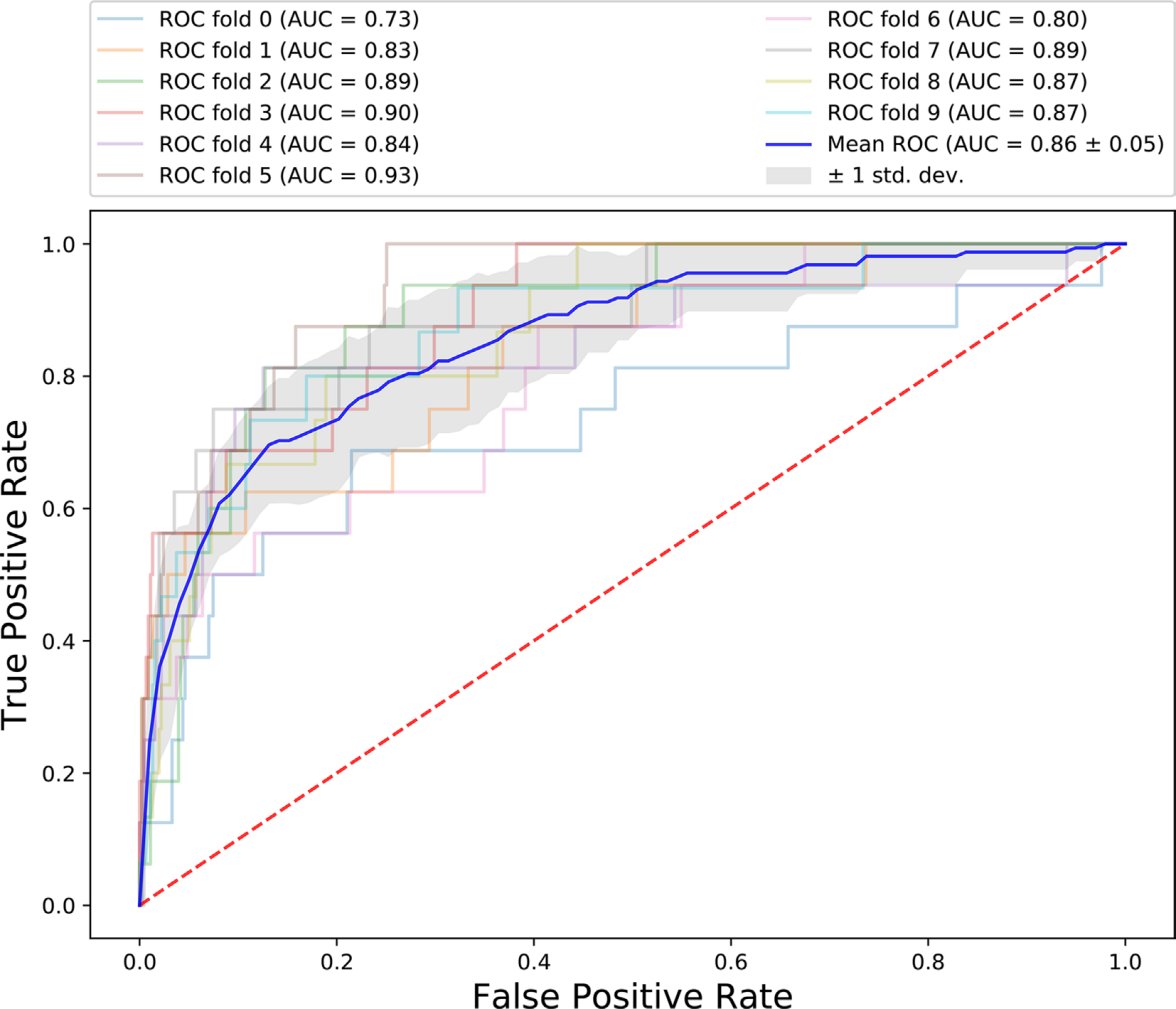
ROC for 10-fold cross-validation.

The results of 10-fold cross-validation are presented in **Figure 3**. We drew 10 ROC curves, which had minimum and maximum AUC values of 0.73 and 0.93, respectively, and an average value of 0.86 ± 0.6. In addition to AUC values, additional evaluation indicators were used to assess our results, including precision, recall, and F1-Score (**Table 2**). The average precision, recall, and F1-Score values were 0.72, 0.62, and 0.65, respectively. Overall, these data demonstrate that CRlncRC2 is an efficient tool for identification of lncRNAs related with cancer, with high accuracy and stable performance.

**Table 2:** Performance of 10-fold cross-validation.

Ten-fold cross-validation	Precision	Recall	F1-Score
macro avg fold 0	0.74	0.56	0.59
macro avg fold 1	0.90	0.66	0.72
macro avg fold 2	0.57	0.55	0.56
macro avg fold 3	0.77	0.62	0.67
macro avg fold 4	0.71	0.62	0.65
macro avg fold 5	0.78	0.71	0.74
macro avg fold 6	0.67	0.65	0.66
macro avg fold 7	0.74	0.65	0.68
macro avg fold 8	0.65	0.59	0.62
macro avg fold 9	0.70	0.60	0.63

**Figure 4:**
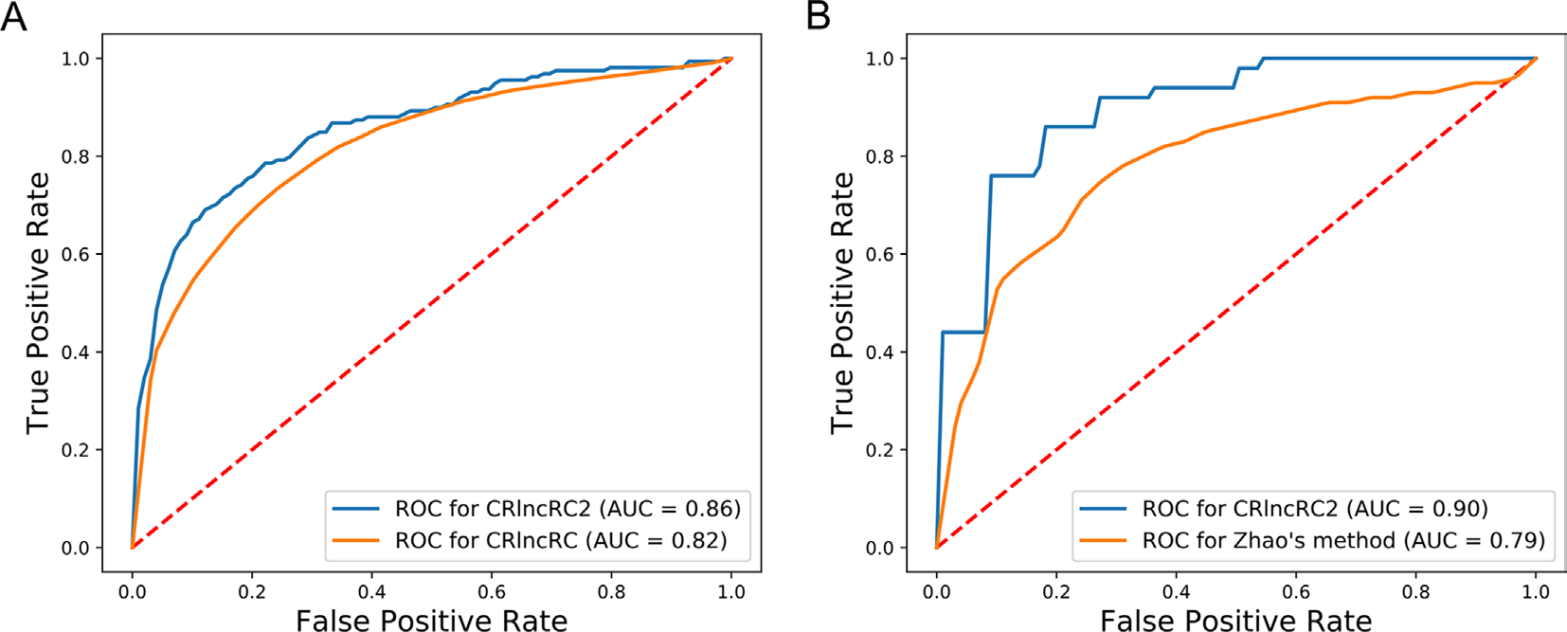
Comparison of accuracy. **(A)** CRlncRC2 ROC generates an AUC value 0.04 higher than that achieved using CRlncRC. **(B)** CRlncRC2 ROC generates an AUC value 0.11 higher than that achieved using the method of Zhao et al.

Compared with other methods, CRlncRC2 has superior performance. Relative to CRlncRC, CRlncRC2 reduced features number from 85 to 51 and the mean AUC value reached 0.86, which is 0.04 higher than that achieved using CRlncRC (**Figure 4A**). Further, we compared the prediction performance of CRlncRC2 with that of the method described by Zhao et al. (2015). To ensure a fair comparison, we retrained our CRlncRC2 method using the dataset reported by Zhao et al. Compared with the method of Zhao et al., the resulting mean AUC value for CRlncRC2 was much higher (0.90 vs. 0.79) (**Figure 4B**).

**Figure 5:**
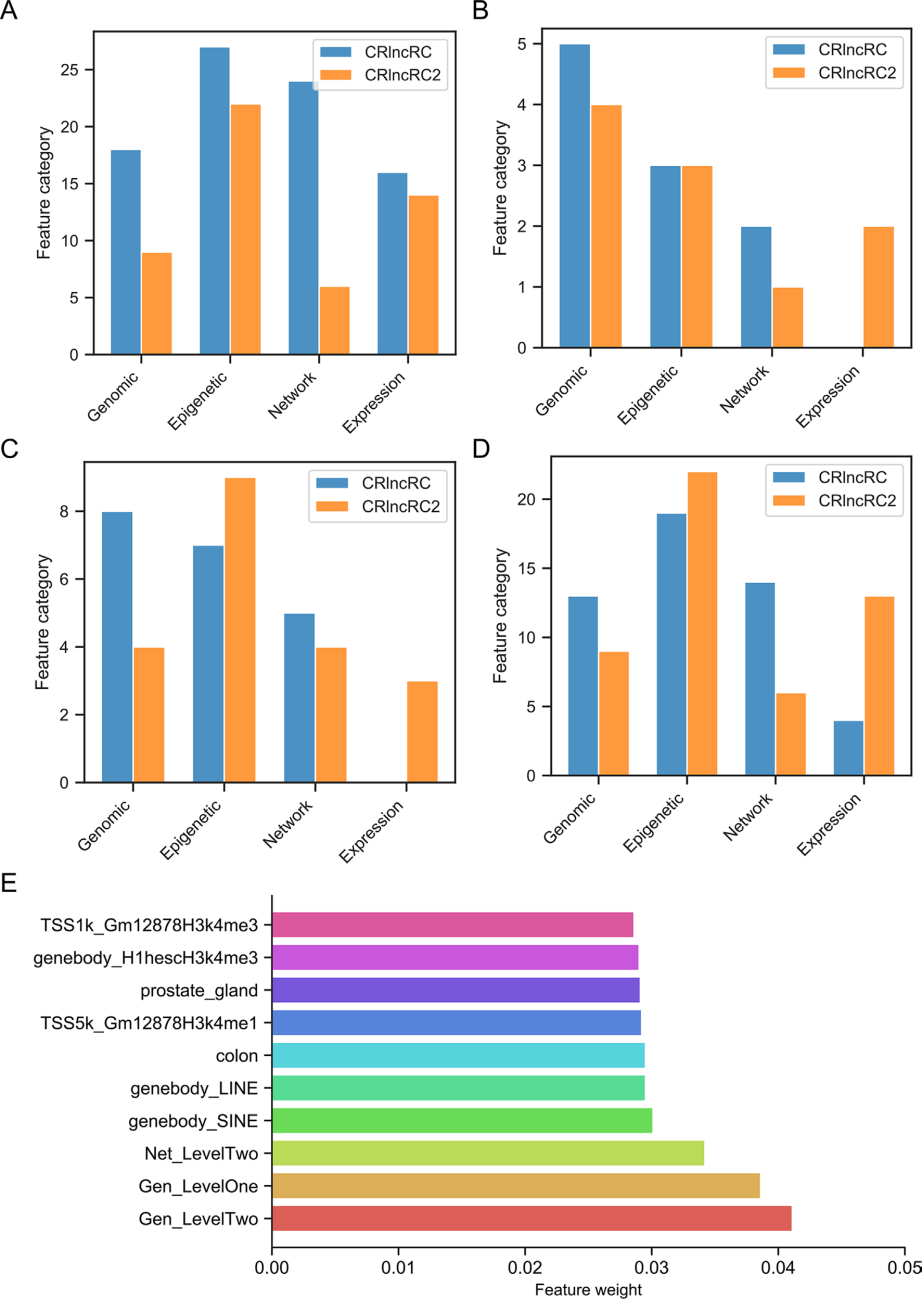
Comparison of feature numbers in the four feature categories. **(A)** Comparison of total features in CRlncRC2 and CRlncRC. CRlncRC contains 85 features, and after feature selection, 51 remained in CRlncRC2. **(B)** Comparison of the top 10 features in CRlncRC2 and CRlncRC. **(C)** Comparison of the top 20 features in CRlncRC2 and CRlncRC. **(D)** Comparison of the top 50 features in CRlncRC2 and CRlncRC. **(E)** Bar plot of the top 10 features used in CRlncRC2.

To determine why CRlncRC2 performed better than CRlncRC, we analyzed the feature importance (XGBoost importance weight) in CRlncRC2 (**Data Sheet 6**). Compared with the features used in CRlncRC, it is clear that the epigenetic and expression feature numbers in CRlncRC2 were almost unchanged, while those of genomic features were reduced by half, while network features were decreased by two thirds (**Figure 5A**). Expression features were two among the top ten most important features in CRlncRC2, while they were not among the top ten in CRlncRC (**Figure 5B**). In addition, there are four types of features in the top 20 features of CRlncRC2, indicating that CRlncRC2 can make better use of different features (**Figure 5C**). Furthermore, as illustrated in **Figures 5C, D**, the proportions of epigenetic features among the first 20 and 50 features for CRlncRC2 were much larger than those for CRlncRC. Surprisingly, although genomic and network features accounted for a small proportion, the three synthetic genomic and network features (Gen_LevelTwo, Gen_LevelOne, and Net_LevelTwo) ranked the highest, indicating that synthetic features generated by combining low Laplacian score features may contribute substantially to the model (**Figure 5E**). Two repeat features, short interspersed nuclear element (SINE) and long interspersed nuclear element (LINE) signals on gene bodies, ranked No. 4 and No. 5, followed by gene expression level in colon tissue (No. 6), prostate gland (No. 8), “H3k4me1” epigenetic modification signals within the Transcription Start Site upstream and downstream 5k (TSS5k) region in GM12878 (No. 7), and “H3k4me3” epigenetic modification signals within lncRNA gene body/TSS1k region in H1hesc/GM12878 cell line (No. 9 and No. 10).

**Figure 6:**
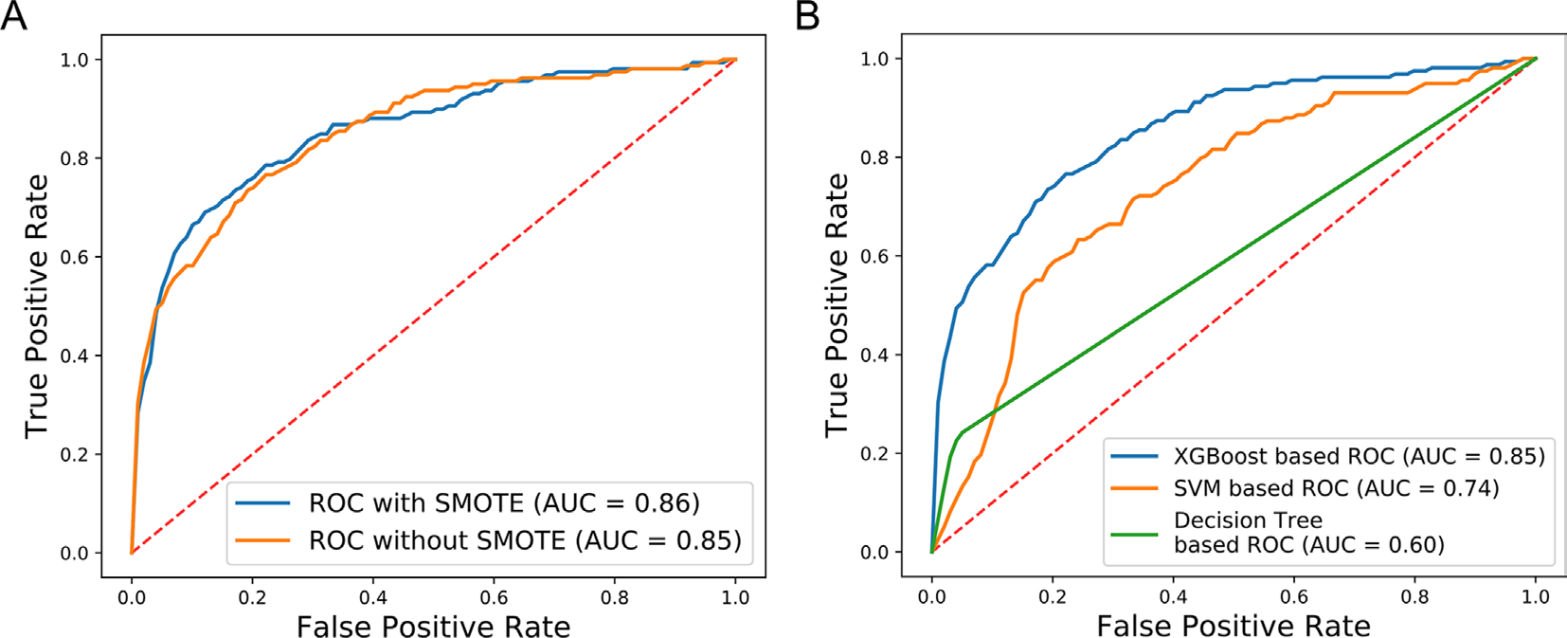
Comparison of SMOTE and non-SMOTE, non-SMOTE XGBoost, and others. **(A)** The ROC generated using SMOTE XGBoost has AUC value 0.01 higher than that achieved using non-SMOTE XGBoost. **(B)** The XGBoost-based ROC without SMOTE generated AUC values 0.11 and 0.25 higher than the SVM-based and Decision Tree-based ROC curves, respectively.

We further evaluated the effectiveness of our approach to dealing with the available imbalanced data. The SMOTE over-sampling method was used to balance the imbalanced data, and it contributed to an increase of 0.01 in the AUC value, relative to non-SMOTE adjusted data (**Figure 6A**). In addition, to compare the performance of different machine learning algorithms, several models were compared using the non-SMOTE adjusted over-sampling data. ROC curve analysis showed that the XGBoost-based method performed better than Decision tree (DT) (0.85 vs. 0.60) and Support Vector Machine (SVM) (0.85 vs. 0.74) -based approaches (**Figure 6B**). These results indicate that our new method facilitated superior performance relative to previous methods. XGBoost contributed substantially to the AUC values, while data over-sampling was also very important.

### Statistical Analysis of Candidate Cancer-Related lncRNA Candidates

**Figure 7:**
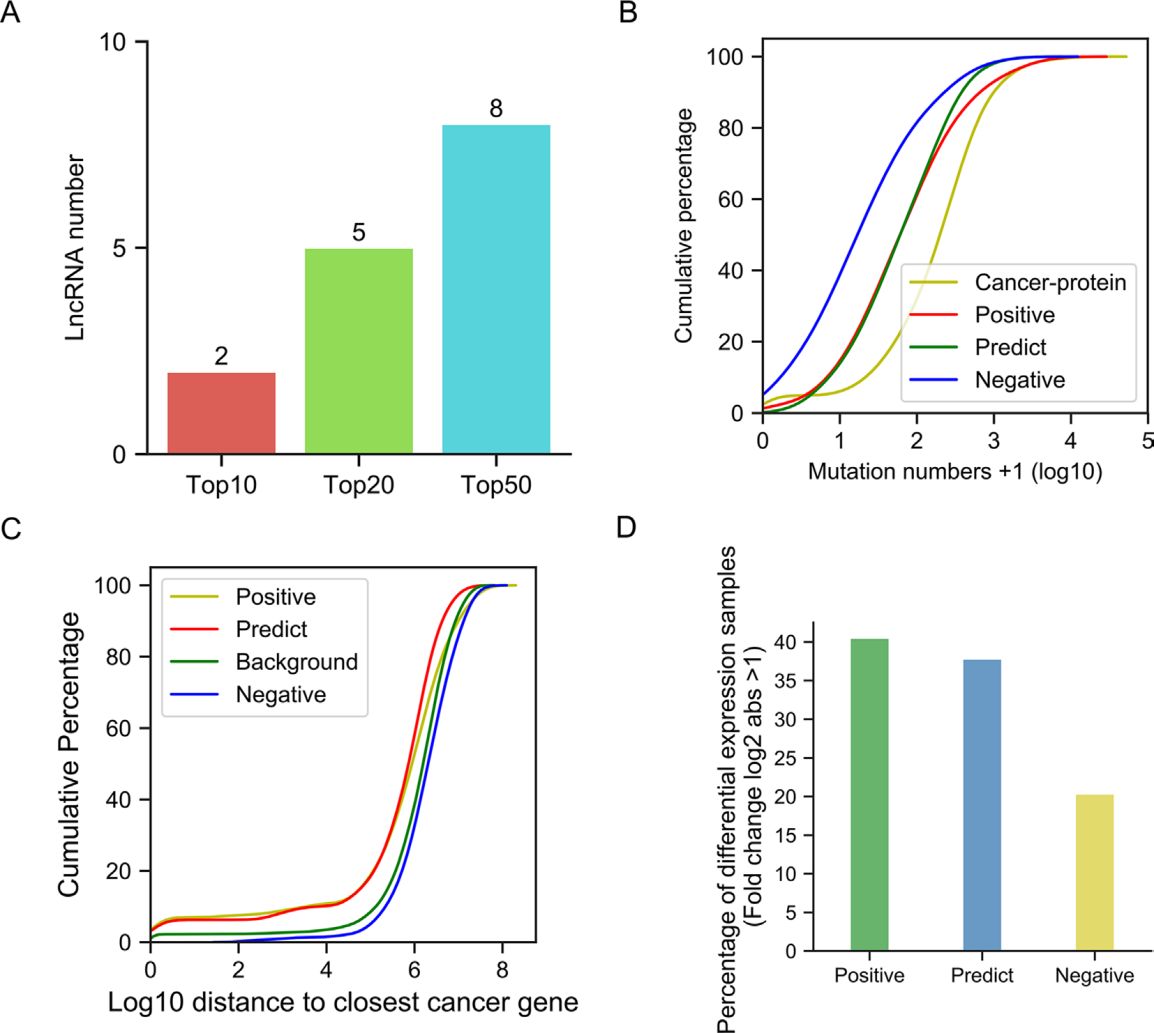
Validation of our predictions. **(A)** Bar plots of cancer-related lncRNA numbers confirmed by Lnc2Cancer in the top 10, top 20, and top 50. **(B)** Cumulative distribution of mutation number. **(C)** Cumulative distribution of the closest distance to cancer-related proteins. (D) Bar plot of the percentage of differentially expressed lncRNAs.

We used the pre-trained model to predict novel candidate cancer-related lncRNAs from 7,253 unknown lncRNAs, which were not in our training or testing sets. Finally, we predicted 439 cancer-related lncRNA candidates (**Data Sheet 7**). First, we used the data from the newly updated database, Lnc2Cancer v2.0, to test our predictions, since we did not collect our positive dataset from this database. We studied the intersection of our predictions and their collections. Among our top 10, 20, and 50 predictions, 2, 5, 8 lncRNAs, respectively, were also collected by Lnc2Cancer, and were functionally validated as cancer-related (**Figure 7A**). In total, 47 candidate cancer-related lncRNAs were found in Lnc2Cancer (**Data Sheet 7**). According to the tag information provided in the Lnc2Cancer database, these lncRNAs can be classified into several categories: drug-resistant, methylation, circulating, transcription factor (TF), and variant (**Data Sheet 8, Figure A**). Further, we selected the top 10 among these 47 cancer-related lncRNAs and evaluated their expression in cancers. Surprisingly, almost all lncRNAs exhibited inconsistent changes in expression in various tissues (**Data Sheet 8, Figure B**), confirming their functional diversity and reflecting the strong tissue specificity of lncRNAs. In addition, the 47 predicted lncRNAs had roles in numerous malignant tumors, including 17 involved in colorectal cancer, 10 in gastric cancer, and 10 in hepatocellular carcinoma (**Data Sheet 8, Figure C**).

Using statistical methods and multigroup data, we further analyzed the reliability of our predictions. First, we hypothesized that the potential cancer-related lncRNAs were likely to have more somatic mutations in cancer genomes, since many previous studies have demonstrated that mutations in functional genes are a primary cause of carcinogenesis. To validate this assumption, we compared the number of somatic mutations (documented in COSMIC) between different lncRNA sets and a cancer-related protein-coding gene set (**Figure 7B**). The results showed that the cancer-related protein-coding gene set, as the positive control, contained far more somatic mutations than the cancer-unrelated lncRNA set (negative control, Kolmogorov-Smirnov test, p-value = 6.10e-33). The somatic mutation numbers in both the positive and predicted cancer-related lncRNA sets were between those of cancer-unrelated lncRNAs and cancer-related protein-coding genes, with a significantly higher quantity than those in cancer-unrelated lncRNAs (Kolmogorov-Smirnov test, p-value 2.35e-07 and 8.27e-06, respectively).

As a number of lncRNAs exert their function in cis, by influencing neighboring genes, we assumed that these potential cancer-related lncRNAs were likely closer to cancer-related protein-coding genes than cancer-unrelated lncRNAs. Therefore, we calculated the distances of different lncRNA sets to their closest cancer-related proteins, and compared them with the random background (i.e., the distance between cancer-related protein-coding genes and random positions in genome) (**Figure 7C**). We found that the distances between cancer-unrelated lncRNAs and cancer-related protein-coding genes were significantly larger than those between cancer-related lncRNAs and cancer-related protein-coding genes (Kolmogorov-Smirnov test, p-value = 4.1e-4). Similarly, the distance of predicted cancer-related lncRNAs from cancer-related protein-coding genes was far shorter than that of cancer-unrelated lncRNAs (Kolmogorov-Smirnov test, p-value = 4.9e-06). Moreover, no significant difference in distance was detected between background and the cancer-unrelated lncRNA set, as expected.

Next, we examined whether the expression levels of cancer-related lncRNAs differed from those of cancer-unrelated lncRNAs in cancer samples (**Figure 7D**). Using lncRNA expression data from the TANRIC database, we calculated the percentage of lncRNAs that were differentially expressed (absolute log_2_-fold change > 1) between cancer and paracancerous tissue sample pairs, to determine whether this differed among the lncRNA sets. We found that lncRNAs in the positive set had the highest percentage of differentially expressed genes (approximately 40%), while the value for the negative set was only approximately 20%. Among predicted cancer-related lncRNAs, > 35% of them showed differential expression. These results further support the association of our prediction products with cancer, and also reveal that simple dependence on differential expression to identify cancer-related lncRNAs is far from sufficient.

### Case Study

**Figure 8:**
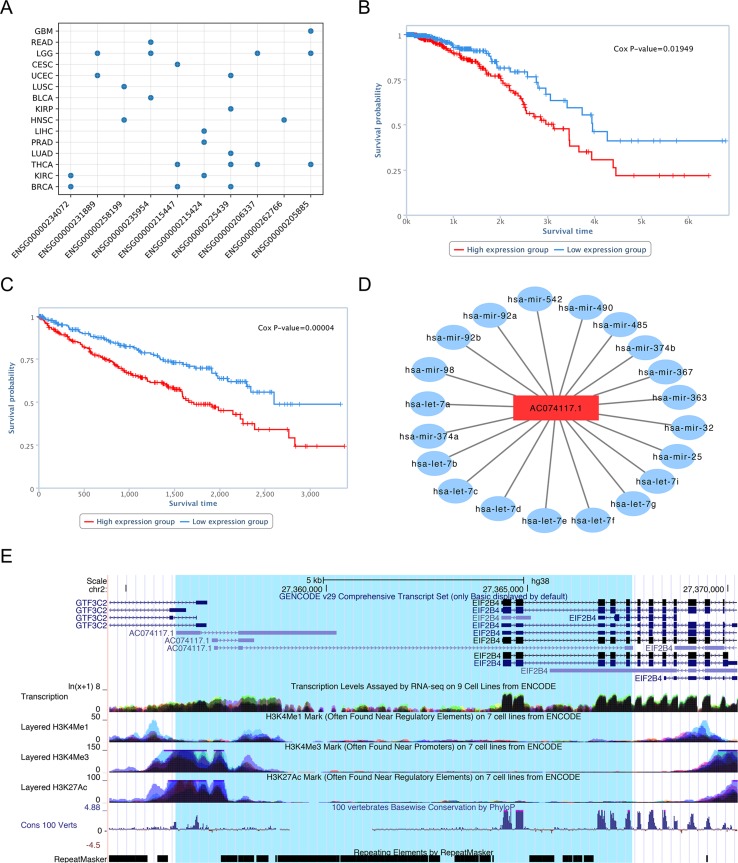
Characterization of lncRNA AC074117.1. **(A)** Survival statistics for the top 10 lncRNA predictions. **(B)** Survival analysis of AC07117.1 in BRCA. **(C)** Survival analysis of AC07117.1 in KIRC. **(D)** Sub-network of AC07117.1 and cancer-related miRNAs. **(E)** Gene structure, epigenetic features, conservation, and repeats of AC07117.1 in the UCSC genome browser.

Although functional identification of lncRNAs is very challenging, using bioinformatics analysis, database searches, and literature review, we can uncover evidence that our predictions represent lncRNAs with functions in cancer. For the top 10 candidate genes we used the TANRIC database to generate Kaplan-Meier survival curves for each cancer type. The results showed that there was a significant difference in the overall survival rate between low and high lncRNA expression groups for all genes in at least one tumor tissues (**Figure 8A**).

For example, survival analysis of the No. 1 lncRNA, AC074117.1, indicated significant differences in survival time between low and high expression groups in individuals with invasive breast carcinoma (BRCA) and kidney renal clear cell carcinoma (KIRC), with p-values of 1.5e-2 and 4.0e-5, respectively (**Figure 8B, C**). To study the regulatory function of AC074117.1, we downloaded data on cancer-related small RNA molecules from The Human microRNA Disease Database (HMDD) (Huang et al., 2019), and the interaction network between lncRNAs and miRNAs from StarBase (Li et al., 2014). Subsequently, we constructed an interaction network between AC074117.1 and cancer-related microRNAs (**Figure 8D**). In addition, according to predictions using the LncRNA and Disease Database (version 2.0), AC074117.1 likely targets a gene cluster on chromosome 2, and is associated with a variety of cancers (Bao et al., 2019). Together, all these clues suggest that AC074117.1 may be involved in cancer and act as a ceRNA. As shown in **Figure 8E** (data from the UCSC genome browser), AC074117.1 is highly expressed in almost all tissues. Further, there is histone methylation signal in the AC074117.1 transcription start site. The H3K4Me3 and H3K27Ac signals in the first exon were high, while the H3K4Me1 signals were relatively weak. Moreover, high conservation signals (100 vertebrates basewise conservation scores generated using PlyloP) were found in its exon regions. Notably, there are a large number of repetitive elements in the whole body region of AC074117.1. These methylation signals and repeat elements may contribute to the mechanism by which this lncRNA is involved in cancer progression (Anwar et al., 2017; Di Ruocco et al., 2018; Solovyov et al., 2018). In conclusion, our predictions indicate that lncRNA AC074117.1 has a strong potential correlation with cancer.

In addition, recent literature reports support some of the predicted lncRNAs in the top 10 list; for example, the *TRAF3IP2-AS1* lncRNA ranked second (No. 2) among our predictions and is a hub gene in a lncRNA-mediated ceRNA network that competes with the onco-lncRNAs, PVT1 and XIST, and could be a clinically relevant biomarker in glioblastoma (Zan and Li, 2019). TTC28-AS1 (No. 4) is an antisense RNA of TTC28 which is associated with colorectal cancer (Pitkanen et al., 2014). Further, C1RL-AS1 (No. 10) has been linked to angiogenesis, as predicted in the ANGIOGENES database (Muller et al., 2016).

## Discussion

Accumulating reports demonstrate that lncRNAs have significant roles in human cancers. Using experimental methods to study the relationships between lncRNA and cancer is time consuming and costly. In contrast, computational methods enable integration of multi-omics data and provide additional information for data mining. In this study, we developed a new method, CRlncRC2, based on a powerful machine learning algorithm — XGBoost, Laplacian score feature selection, and SMOTE over-sampling, to predict associations of lncRNAs with cancer. Compared with CRlncRC, CRlncRC2 improves the performance while requires fewer features (see **Table 3** for a detailed comparison). The results show that CRlncRC2 is much more sensitive and specific than the previous version (CRlncRC), primarily due to the selected algorithm model, as the results show huge differences between results generated using other methods and those from application of XGBoost. XGBoost has also been used in numerous other projects, achieving good results. For example, Zheng et al. developed a scalable, flexible approach, BiXGBoost, to reconstruct gene regulatory networks (GRNs), and tested it on DREAM4 and *Escherichia coli* datasets, demonstrating good performance of BiXGBoost in different scale networks (Zheng et al., 2018).

**Table 3:** Comparison of CRlncRC and CRlncRC2.

Method	Algorithm model	Number of Features	Feature selection	Sampling strategy	AUC
CRlncRC	Random Forest	85	No	Under-sampling	0.82
CRlncRC2	XGBoost	51	Yes	Over-sampling	0.86

Machine learning algorithms have important roles in bioinformatics, where they facilitate the solution of problems, such as classification, clustering, regression, and prediction; however, the machine learning approach still faces a number of obstacles in predicting cancer-related lncRNAs. First, for biological data, frequently, only small positive sets are available, due to the difficulty of collecting information, such as patient data and experimental verification of functional genes, which greatly impedes the practical application of machine learning. Further, machine learning models require optimization for high performance, according to the specific data and situation. To address these problems, in this study, we selected the most stringent criteria to select the positive and negative sets, and used the latest histological information for feature extraction. We chose over-sampling in our new algorithm because it enables use of more information relative to under-sampling, and the results confirmed that it can improve accuracy and specificity. Moreover, we merged features with high Laplacian scores and got eight synthesis features, which had a highest feature importance rank. Our findings suggest that high Laplacian score features still contain useful information and is not good practice to simply discard them.

LncRNAs have been applied in clinical practice as new biomarkers and prognostic indicators. Research on the relationships between lncRNAs and cancer is attractive and progressing very rapidly. Machine learning methods have the power to discover novel lncRNAs, including disease associated lncRNAs (Kang et al., 2017; Bao et al., 2019). Efforts should continue to improve the ability of machine learning algorithms to predict cancer associations. Moreover, with increasing research into lncRNAs, greater quantities of relevant high-throughput data are becoming easier to obtain. The development of functional research into lncRNAs has revealed additional functional elements and mechanisms (Zhang et al., 2014; Brockdorff, 2018). Further, numerous new tools for evaluating the similarity of non-linear sequences, using k-mer content (Kirk et al., 2018) and a new evolutionary classification perspective (Chen et al., 2016), have been developed, which can be used to extract new features, such as lncRNA conservation. These can facilitate better application of bioinformatics methods to predict cancer-related lncRNAs and assist in cancer diagnosis and treatment.

## Conclusions

In this study, we upgraded CRlncRC to CRlncRC2, using a powerful machine learning algorithm (XGBoost), Laplacian score feature selection, and an advanced over-sampling method (SMOTE). The results show that both XGBoost and SMOTE can help to improve model accuracy and specificity. After feature engineering, most of the expressed and methylated features are retained, indicating their importance for predicting lncRNAs with potential functions in cancer. Using much fewer features, CRlncRC2 has a mean AUC value 0.04 higher than that of CRlncRC. In addition, our predicted top-ranking cancer-related lncRNA candidates are supported by Inc2Cancer v2.0, literature reports, and statistical data. In summary, CRlncRC2 is an effective and useful method for lncRNA-cancer association identification.

## Data Availability

The datasets analyzed for this study can be found at https://github.com/xuanblo/CRlncRC2.

## Author Contributions

CL and LC conceived, designed, and supervised this study. JW and XZ collected and compiled data from the literature and public databases. XZ, TL, and JW designed and developed the data analysis. JL participated in discussion of the project. XZ, JW, TJ, and CL compiled the manuscript draft. CL, LC, and JL revised the manuscript. All authors reviewed, edited, and approved the manuscript.

## Funding

This work was supported by the National Natural Science Foundation of China (No. 31471220, 91440113), Start-up Fund from Xishuangbanna Tropical Botanical Garden, ”Top Talents Program in Science and Technology” from Yunnan Province, Science and Technology Development Fund, Macau S.A.R. (097/2015/A3, 196/2017/A3).

## Conflict of Interest Statement

The authors declare that the research was conducted in the absence of any commercial or financial relationships that could be construed as a potential conflict of interest.

## Abbreviations

AUC, area under the ROC curve; ceRNA, competing endogenous RNA; DT, decision tree; lncRNA, long non-coding RNA; ROC, Receiver operating characteristic; SVM, support vector machines; XGBoost, extreme gradient boosting machine

## Supplementary Material

Data Sheet 1 Positive dataset. XLSX 11KB

Data Sheet 2 Negative dataset. XLSX 66KB

Data Sheet 3 Feature categories. DOCX 16KB

Data Sheet 4 Bigtable. CSV 6.9M

Data Sheet 5 Cumulative curves of positive and negative lncRNAs for all features. PDF 538KB

Data Sheet 6 Feature importance. CSV 1KB

Data Sheet 7 Predicted positives. XLSX 27KB

Data Sheet 8 Statistics of the interactions with Lnc2Cancer v2.0. PDF 207KB

## References

[B1] Aab A. Abreu P. Aglietta M. Ahn E. J. Al Samarai I. Albuquerque I. F. (2016). Measurement of the radiation energy in the radio signal of extensive air showers as a universal estimator of cosmic-ray energy. Phys. Rev. Lett. 116, 241101. 10.1103/PhysRevLett.116.241101 27367377

[B2] Anwar S. L. Wulaningsih W. Lehmann U. (2017). Transposable elements in human cancer: causes and consequences of deregulation. Int. J. Mol. Sci. 18 (5), 974. 10.3390/ijms18050974 PMC5454887 28471386

[B3] Balas M. M. Johnson A. M. (2018). Exploring the mechanisms behind long noncoding RNAs and cancer. Noncoding RNA Res. 3, 108–117. 10.1016/j.ncrna.2018.03.001 30175284 PMC6114262

[B4] Bao Z. Yang Z. Huang Z. Zhou Y. Cui Q. Dong D. (2019). LncRNADisease 2.0: an updated database of long non-coding RNA-associated diseases. Nucleic Acids Res. 47, D1034–D1037. 10.1093/nar/gky905 30285109 PMC6324086

[B5] Bhan A. Mandal S. S. (2015). LncRNA HOTAIR: a master regulator of chromatin dynamics and cancer. Biochim. Biophys. Acta 1856, 151–164. 10.1016/j.bbcan.2015.07.001 26208723 PMC4544839

[B6] Bhan A. Soleimani M. Mandal S. S. (2017). Long noncoding RNA and cancer: a new paradigm. Cancer Res. 77, 3965–3981. 10.1158/0008-5472.CAN-16-2634 28701486 PMC8330958

[B7] Brockdorff N. (2018). Local tandem repeat expansion in Xist RNA as a Model for the Functionalisation of ncRNA. Non-coding RNA, 4 (4), 28. 10.3390/ncrna4040028 PMC6316617 30347781

[B8] Chen J. Shishkin A. A. Zhu X. Kadri S. Maza I. Guttman M. (2016). Evolutionary analysis across mammals reveals distinct classes of long non-coding RNAs. Genome Biol. 17, 19. 10.1186/s13059-016-0880-9 26838501 PMC4739325

[B9] Chen T. Guestrin C. (2016). “XGBoost: A Scalable Tree Boosting System,” in Proceedings of the 22nd ACM SIGKDD International Conference on Knowledge Discovery and Data Mining. (San Francisco, California, USA: ACM). 10.1145/2939672.2939785

[B10] Chung F. R. K. (1997). Spectral Graph Theory. United States: American Mathematical Society.

[B11] De Kok J. B. Verhaegh G. W. Roelofs R. W. Hessels D. Kiemeney L. A. Aalders T. W. (2002). DD3(PCA3), a very sensitive and specific marker to detect prostate tumors. Cancer Res. 62, 2695–2698. 11980670

[B12] Di Ruocco F. Basso V. Rivoire M. Mehlen P. Ambati J. De Falco S. (2018). Alu RNA accumulation induces epithelial-to-mesenchymal transition by modulating miR-566 and is associated with cancer progression. Oncogene 37, 627–637. 10.1038/onc.2017.369 28991230 PMC5799714

[B13] Gao Y. Wang P. Wang Y. Ma X. Zhi H. Zhou D. (2019). Lnc2Cancer v2.0: updated database of experimentally supported long non-coding RNAs in human cancers. Nucleic Acids Res. 47, D1028–D1033. 10.1093/nar/gky1096 30407549 PMC6324001

[B14] Hanahan D. Weinberg R. A. (2011). Hallmarks of cancer: the next generation. Cell 144, 646–674. 10.1016/j.cell.2011.02.013 21376230

[B15] He X. Cai D. Niyogi P. (2005). “Laplacian score for feature selection,” in Proceedings of the 18th International Conference on Neural Information Processing Systems. (Vancouver, British Columbia, Canada: MIT Press).

[B16] He X. Niyogi P. (2003). “Locality Preserving Projections,” in Proceedings of the 16th International Conference on Neural Information Processing Systems (Whistler, British Columbia, Canada: MIT Press).

[B17] Hessels D. Klein Gunnewiek J. M. Van Oort I. Karthaus H. F. Van Leenders G. J. Van Balken B. (2003). DD3(PCA3)-based molecular urine analysis for the diagnosis of prostate cancer. Eur. Urol. 44, 8–15 discussion 15–16. 10.1016/S0302-2838(03)00201-X 12814669

[B18] Huang Z. Shi J. Gao Y. Cui C. Zhang S. Li J. (2019). HMDD v3.0: a database for experimentally supported human microRNA-disease associations. Nucleic Acids Res. 47, D1013–D1017. 10.1093/nar/gky1010 30364956 PMC6323994

[B19] Kang Y. J. Yang D. C. Kong L. Hou M. Meng Y. Q. Wei L. (2017). CPC2: a fast and accurate coding potential calculator based on sequence intrinsic features. Nucleic Acids Res. 45, W12–W16. 10.1093/nar/gkx428 28521017 PMC5793834

[B20] Kanwal R. Gupta S. (2010). Epigenetics and cancer. J. Appl. Physiol. 109 (1985), 598–605. 10.1152/japplphysiol.00066.2010 20203073 PMC2928601

[B21] Kirk J. M. Kim S. O. Inoue K. Smola M. J. Lee D. M. Schertzer M. D. (2018). Functional classification of long non-coding RNAs by k-mer content. Nat. Genet. 50, 1474–1482. 10.1038/s41588-018-0207-8 30224646 PMC6262761

[B22] Kogure T. Yan I. K. Lin W. L. Patel T. (2013). Extracellular Vesicle-Mediated Transfer of a Novel Long Noncoding RNA TUC339: A Mechanism of Intercellular Signaling in Human Hepatocellular Cancer. Genes Cancer 4, 261–272. 10.1177/1947601913499020 24167654 PMC3807642

[B23] Larranaga P. Calvo B. Santana R. Bielza C. Galdiano J. Inza I. (2006). Machine learning in bioinformatics. Brief Bioinform. 7, 86–112. 10.1093/bib/bbk007 16761367

[B24] Li J. Cheng K. Wang S. Morstatter F. Trevino R. P. Tang J. (2017). Feature Selection: A Data Perspective. ACM Comput. Surv. 50, 1–45. 10.1145/3136625

[B25] Li J. H. Liu S. Zhou H. Qu L. H. Yang J. H. (2014). starBase v2.0: decoding miRNA-ceRNA, miRNA-ncRNA and protein-RNA interaction networks from large-scale CLIP-Seq data. Nucleic Acids Res. 42, D92–D97. 10.1093/nar/gkt1248 24297251 PMC3964941

[B26] Mercer T. R. Mattick J. S. (2013). Structure and function of long noncoding RNAs in epigenetic regulation. Nat. Struct. Mol. Biol. 20, 300–307. 10.1038/nsmb.2480 23463315

[B27] Muller R. Weirick T. John D. Militello G. Chen W. Dimmeler S. (2016). ANGIOGENES: knowledge database for protein-coding and noncoding RNA genes in endothelial cells. Sci. Rep. 6, 32475. 10.1038/srep32475 27582018 PMC5007478

[B28] Nakamura M. Kajiwara Y. Otsuka A. Kimura H. (2013). LVQ-SMOTE - learning vector quantization based synthetic minority over-sampling Technique for biomedical data. BioData Min. 6 (1), 16. 24088532 10.1186/1756-0381-6-16 PMC4016036

[B29] Pitkanen E. Cajuso T. Katainen R. Kaasinen E. Valimaki N. Palin K. (2014). Frequent L1 retrotranspositions originating from TTC28 in colorectal cancer. Oncotarget 5, 853–859. 10.18632/oncotarget.1781 24553397 PMC3996660

[B30] Ransohoff J. D. Wei Y. Khavari P. A. (2018). The functions and unique features of long intergenic non-coding RNA. Nat. Rev. Mol. Cell. Biol. 19, 143–157. 10.1038/nrm.2017.104 29138516 PMC5889127

[B31] Renganathan A. Felley-Bosco E. (2017). Long noncoding RNAs in cancer and therapeutic potential. Adv. Exp. Med. Biol. 1008, 199–222. 10.1007/978-981-10-5203-3_7 28815541

[B32] Siegel R. L. Miller K. D. Jemal A. (2018). Cancer statistics, 2018. CA Cancer J. Clin. 68, 7–30. 10.3322/caac.21442 29313949

[B33] Solovyov A. Vabret N. Arora K. S. Snyder A. Funt S. A. Bajorin D. F. (2018). Global cancer transcriptome quantifies repeat element polarization between immunotherapy responsive and T cell suppressive classes. Cell Rep. 23, 512–521. 10.1016/j.celrep.2018.03.042 29642008 PMC6016853

[B34] Tinzl M. Marberger M. Horvath S. Chypre C. (2004). DD3PCA3 RNA analysis in urine–a new perspective for detecting prostate cancer. Eur. Urol. 46, 182–186; discussion 187. 10.1016/j.eururo.2004.06.004 15245811

[B35] Wang J. Zhang X. Chen W. Hu X. Li J. Liu C. (2019). Regulatory roles of long noncoding RNAs implicated in cancer hallmarks. Int. J. Cancer 10.1002/ijc.32277 30873588

[B36] Wang J. Zhang X. Chen W. Li J. Liu C. (2018). CRlncRNA: a manually curated database of cancer-related long non-coding RNAs with experimental proof of functions on clinicopathological and molecular features. BMC Med. Genomics 11, 114. 10.1186/s12920-018-0430-2 30598113 PMC6311896

[B37] Welter D. Macarthur J. Morales J. Burdett T. Hall P. Junkins H. (2014). The NHGRI GWAS Catalog, a curated resource of SNP-trait associations. Nucleic Acids Res. 42, D1001–D1006. 10.1093/nar/gkt1229 24316577 PMC3965119

[B38] Yang W. Hong L. Xu X. Wang Q. Huang J. Jiang L. (2017). LncRNA GAS5 suppresses the tumorigenesis of cervical cancer by downregulating miR-196a and miR-205. Tumour Biol. 39, 1010428317711315. 10.1177/1010428317711315 28671039

[B39] Yang Y. R. Zang S. Z. Zhong C. L. Li Y. X. Zhao S. S. Feng X. J. (2014). Increased expression of the lncRNA PVT1 promotes tumorigenesis in non-small cell lung cancer. Int. J. Clin. Exp. Pathol. 7, 6929–6935. 25400777 PMC4230094

[B40] Yarmishyn A. A. Kurochkin I. V. (2015). Long noncoding RNAs: a potential novel class of cancer biomarkers. Front. Genet. 6, 145. 10.3389/fgene.2015.00145 25954300 PMC4407501

[B41] You J. S. Jones P. A. (2012). Cancer genetics and epigenetics: two sides of the same coin? Cancer Cell 22, 9–20. 10.1016/j.ccr.2012.06.008 22789535 PMC3396881

[B42] Zan X. Y. Li L. (2019). Construction of lncRNA-mediated ceRNA network to reveal clinically relevant lncRNA biomarkers in glioblastomas. Oncol. Lett. 17, 4369–4374. 10.3892/ol.2019.10114 30944630 PMC6444437

[B43] Zhang B. Gunawardane L. Niazi F. Jahanbani F. Chen X. Valadkhan S. (2014). A novel RNA motif mediates the strict nuclear localization of a long noncoding RNA. Mol. Cell Biol. 34, 2318–2329. 10.1128/MCB.01673-13 24732794 PMC4054287

[B44] Zhang X. Wang J. Li J. Chen W. Liu C. (2018). CRlncRC: a machine learning-based method for cancer-related long noncoding RNA identification using integrated features. BMC Med. Genomics 11, 120. 10.1186/s12920-018-0436-9 30598114 PMC6311943

[B45] Zhao T. Xu J. Liu L. Bai J. Xu C. Xiao Y. (2015). Identification of cancer-related lncRNAs through integrating genome, regulome and transcriptome features. Mol. Biosyst. 11, 126–136. 10.1039/C4MB00478G 25354589

[B46] Zheng R. Li M. Chen X. Wu F. X. Pan Y. Wang J. (2018). BiXGBoost: a scalable, flexible boosting based method for reconstructing gene regulatory networks. Bioinformatics. 35 (11), 1893-1900. 10.1093/bioinformatics/bty908 30395189

