# Supplementary material for: Identification of Cancer-Related Long Non-Coding RNAs Using XGBoost With High Accuracy
Source: Front Genet. 2019 Aug 9;10:735. doi: 10.3389/fgene.2019.00735 (PMC6701491; doi:10.3389/fgene.2019.00735)
Supplement: Data Sheet 3 — Feature categories. DOCX 16KB [file DataSheet_3.docx]

| **Feature category** | **Feature name** | **Database** | **Description** |
| --- | --- | --- | --- |
| expression (16) | prostate_gland | The expression profiles of 16 different tissue types were downloaded from the Human Body Map project. | The expression value of lncRNA. |
|  | lung |  |  |
|  | testis |  |  |
|  | thyroid_gland |  |  |
|  | colon |  |  |
|  | lymph_node |  |  |
|  | brain |  |  |
|  | kidney |  |  |
|  | ovary |  |  |
|  | breast |  |  |
|  | liver |  |  |
|  | adipose_tissue |  |  |
|  | adrenal_gland |  |  |
|  | skeletal_muscle_tissue |  |  |
|  | heart |  |  |
|  | leukocyte |  |  |
| epigenetic (27) | genebody_H1hescH3k4me1 | Pre-calculated by the UCSC genome database (https://genome.ucsc.edu) | The histone methylation signal of lncRNA. |
|  | genebody_H1hescH3k4me3 |  |  |
|  | TSS5k_K562H3k4me1 |  |  |
|  | genebody_Gm12878H3k4me1 |  |  |
|  | genebody_Gm12878H3k4me3 |  |  |
|  | TSS1k_K562H3k4me3 |  |  |
|  | genebody_K562H3k4me1 |  |  |
|  | genebody_Gm12878H3k27ac |  |  |
|  | TSS5k_H1hescH3k27ac |  |  |
|  | TSS5k_H1hescH3k4me3 |  |  |
|  | TSS5k_K562H3k27ac |  |  |
|  | TSS5k_Gm12878H3k4me1 |  |  |
|  | TSS5k_Gm12878H3k4me3 |  |  |
|  | genebody_K562H3k27ac |  |  |
|  | TSS5k_K562H3k4me3 |  |  |
|  | genebody_K562H3k4me3 |  |  |
|  | TSS1k_H1hescH3k27ac |  |  |
|  | TSS5k_H1hescH3k4me1 |  |  |
|  | TSS5k_Gm12878H3k27ac |  |  |
|  | genebody_H1hescH3k27ac |  |  |
|  | TSS1k_Gm12878H3k27ac |  |  |
|  | TSS1k_H1hescH3k4me1 |  |  |
|  | TSS1k_Gm12878H3k4me3 |  |  |
|  | TSS1k_Gm12878H3k4me1 |  |  |
|  | TSS1k_H1hescH3k4me3 |  |  |
|  | TSS1k_K562H3k27ac |  |  |
|  | TSS1k_K562H3k4me1 |  |  |
| genomic (18) | TSS5k_gc | Extracted from GENCODE release 25 annotation. | The GC content of lncRNA. |
|  | exon_gc |  |  |
|  | genebody_gc |  |  |
|  | TSS1k_gc |  |  |
|  | intron_gc |  |  |
|  | micropeptide_aa_avg | We obtained the short peptide sequence of each transcript from the LncRNAWiki. | The average length of micropeptide in lncRNA. |
|  | host_miRNAs | We counted the number of miRNAs (obtained from miRBase, version21) residing in the region of each lncRNA. | The number of miRNAs contained in lncRNA. |
|  | exon_phastCons20way | Pre-calculated by the UCSC genome database (https://genome.ucsc.edu) | The conservation signal in lncRNA. |
|  | intron_phastCons20way |  |  |
|  | TSS1k_phastCons20way |  |  |
|  | TSS1k_SINE |  | The number of repeat elements in lncRNA. |
|  | TSS1k_Satellite |  |  |
|  | genebody_SINE |  |  |
|  | genebody_Satellite |  |  |
|  | genebody_LTR |  |  |
|  | genebody_LINE |  |  |
|  | TSS1k_LTR |  |  |
|  | TSS1k_LINE |  |  |
| network (24) | ERBB2 | The expression profiles of 16 different tissue types were downloaded from the Human Body Map project. The cancer-related protein-coding gene list is downloaded from Cancer Gene Census (https://cancer.sanger.ac.uk/census). | The SCC values with Top20 mutational hotspots cancer driver protein-coding genes. |
|  | CTNNB1 |  |  |
|  | CDKN2A |  |  |
|  | SF3B1 |  |  |
|  | APC |  |  |
|  | EGFR |  |  |
|  | FBXW7 |  |  |
|  | RAC1 |  |  |
|  | U2AF1 |  |  |
|  | IDH1 |  |  |
|  | KRAS |  |  |
|  | AKT1 |  |  |
|  | HRAS |  |  |
|  | BRAF |  |  |
|  | NRAS |  |  |
|  | H3F3A |  |  |
|  | TP53 |  |  |
|  | PIK3CA |  |  |
|  | GNAS |  |  |
|  | PTEN |  |  |
|  | hbm_cancer-protein_degree |  | The degree of lncRNA with cancer protein genes. |
|  | hbm_total_degree |  | The total degree of lncRNA in co-expression network. |
|  | cancer-miRNA_targets | The cancer associated miRNA and its target lncRNA were downloaded from HMDD. | The number of cancer associated miRNA targets in lncRNA. |
|  | miRNA_targets | The miRNA and lncRNA interaction data were download from Starbase. | The number of miRNA targets in lncRNA. |
