# Supplementary figures and images for: Identification of Cancer-Related Long Non-Coding RNAs Using XGBoost With High Accuracy
Source: Front Genet. 2019 Aug 9;10:735. doi: 10.3389/fgene.2019.00735 (PMC6701491; doi:10.3389/fgene.2019.00735)

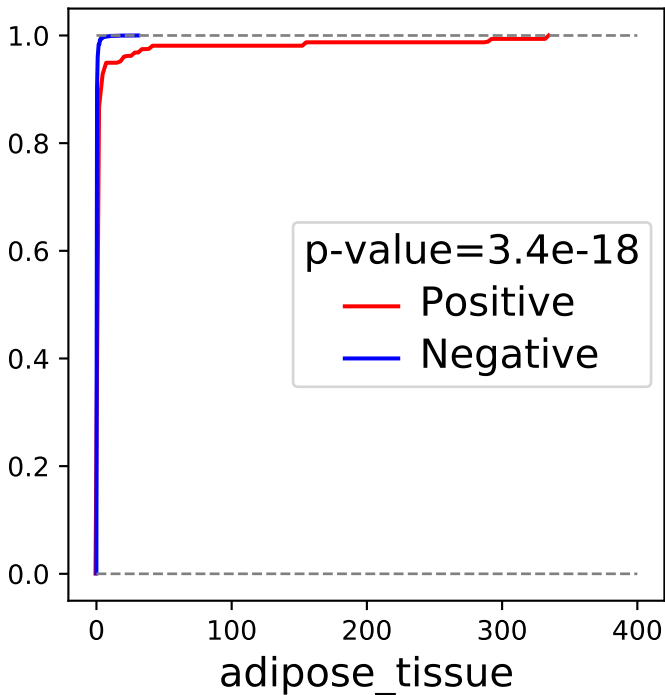

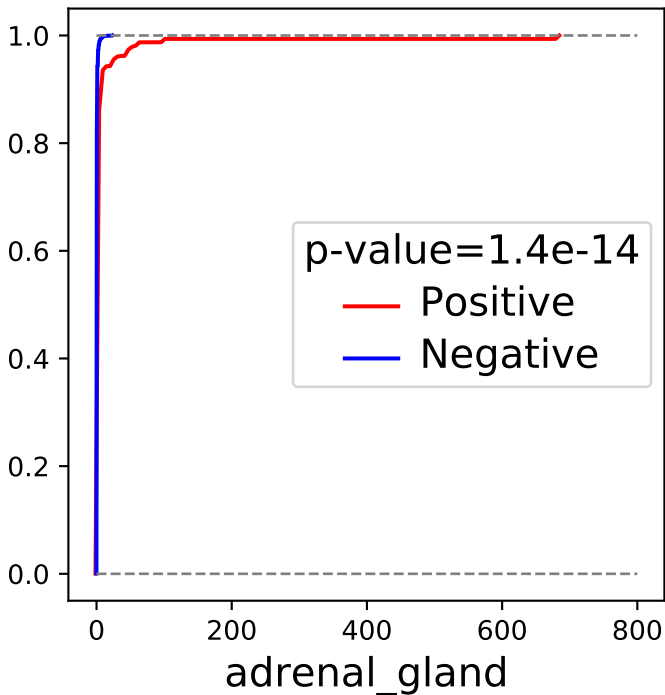

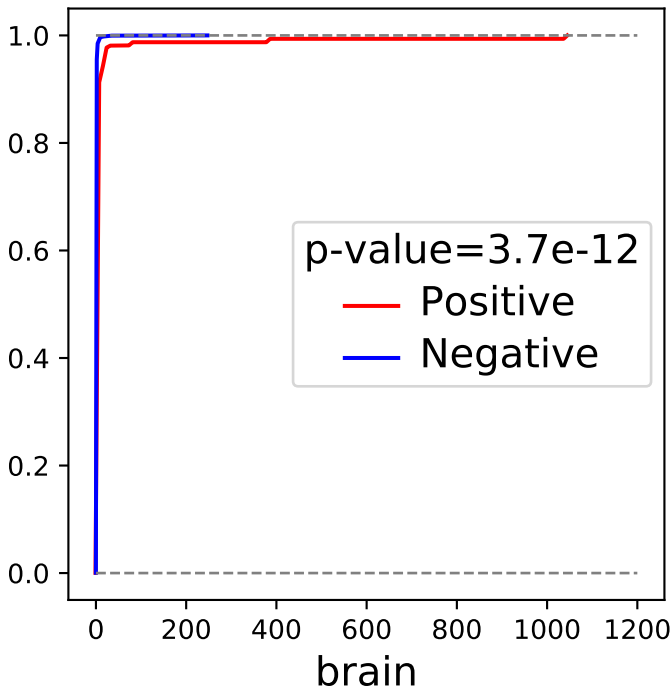

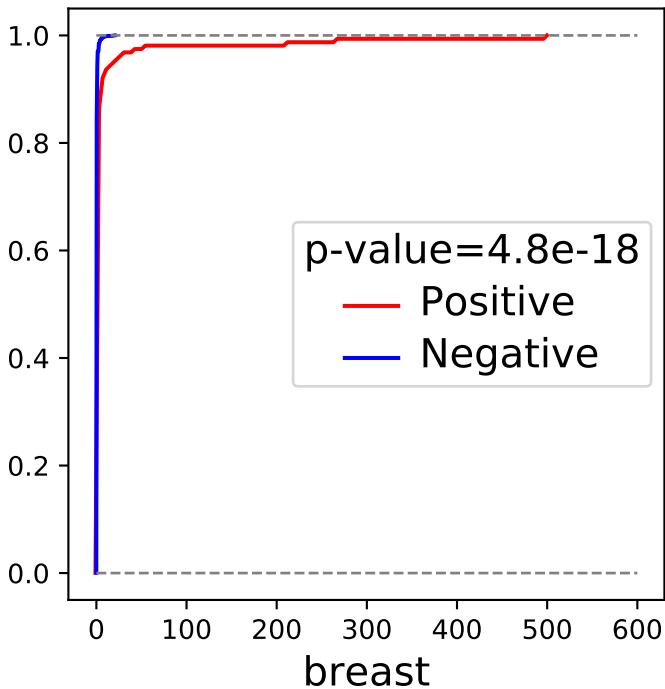

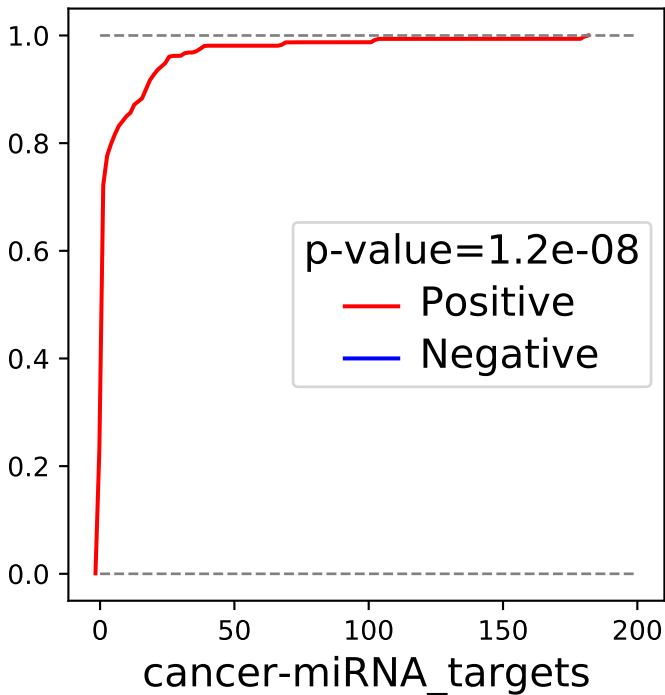

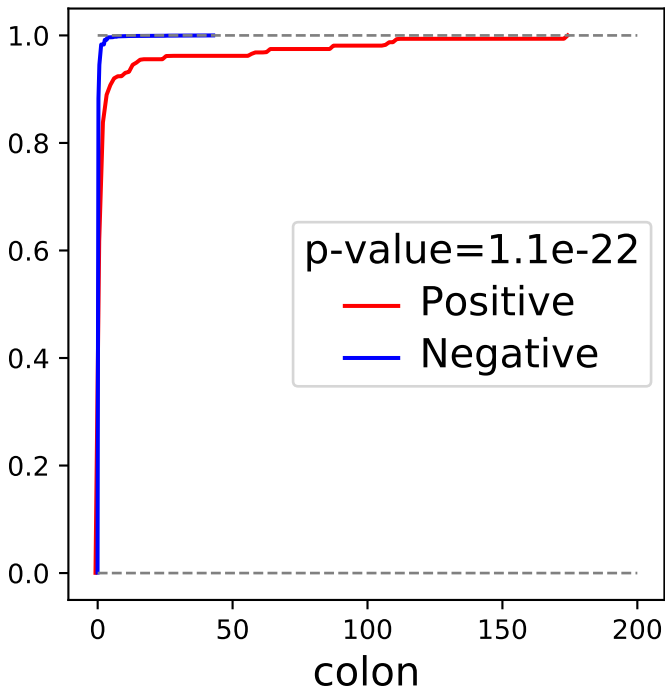

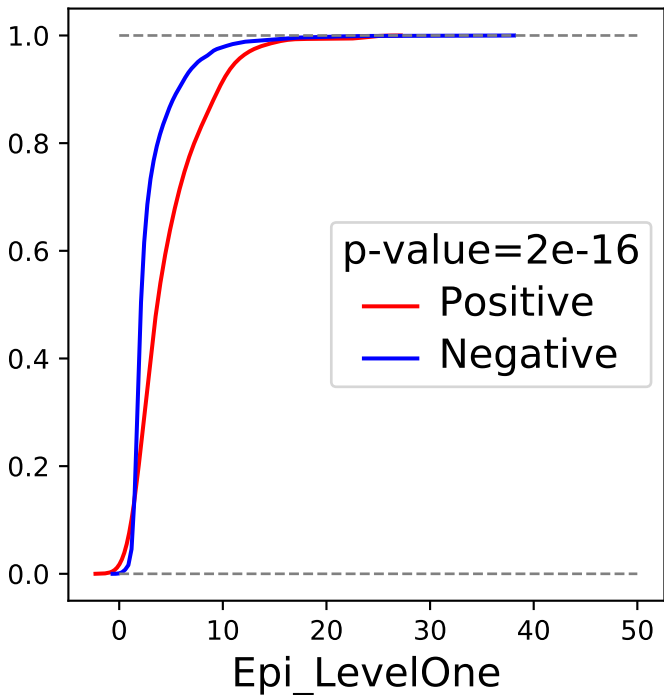

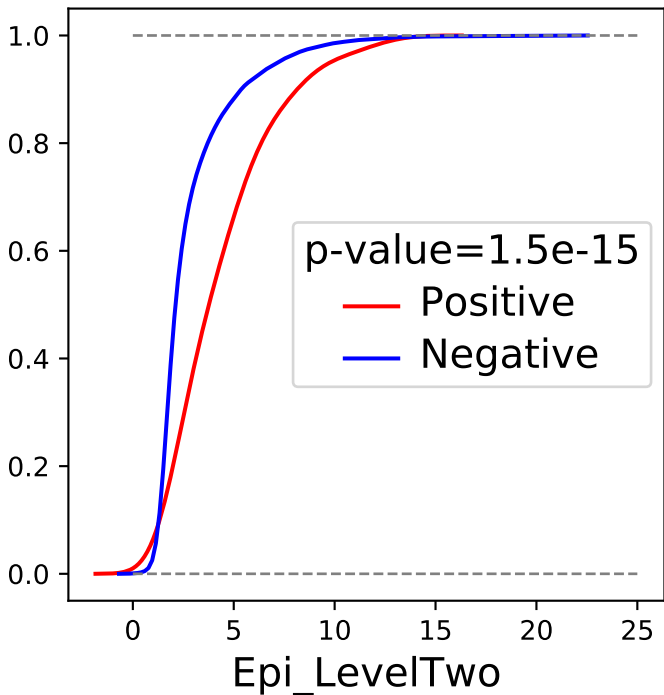

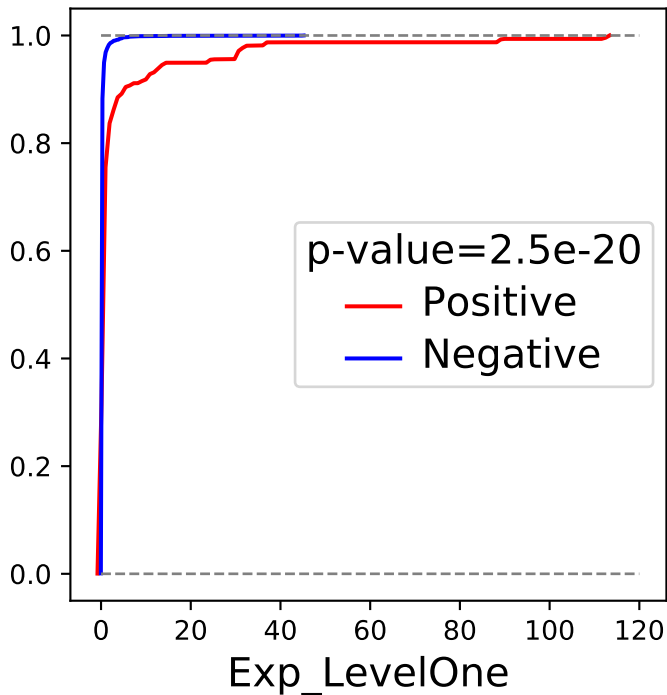

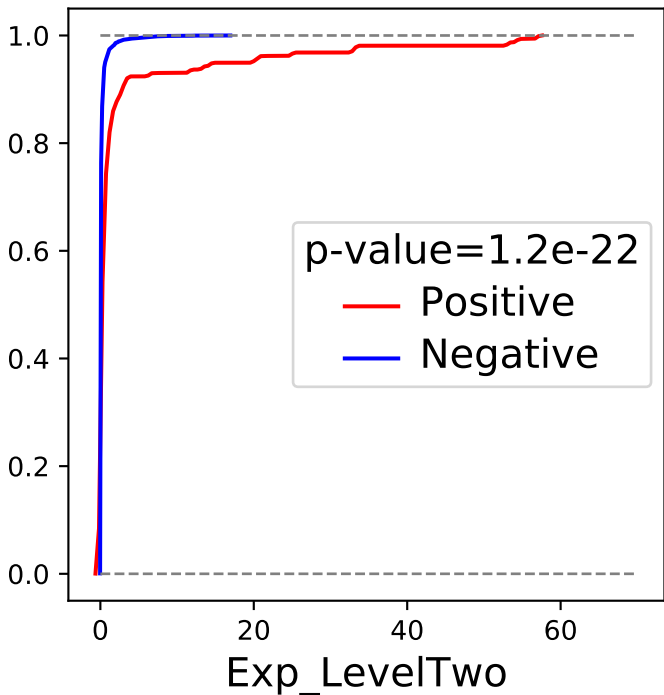

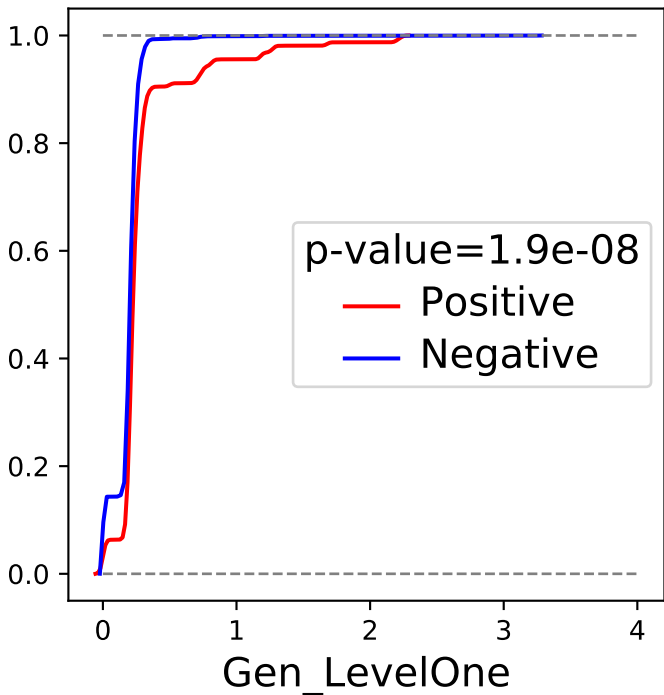

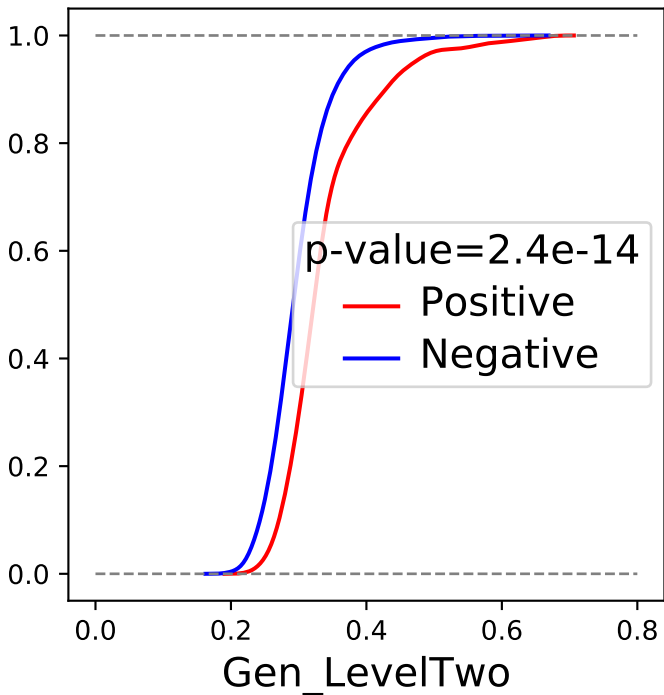

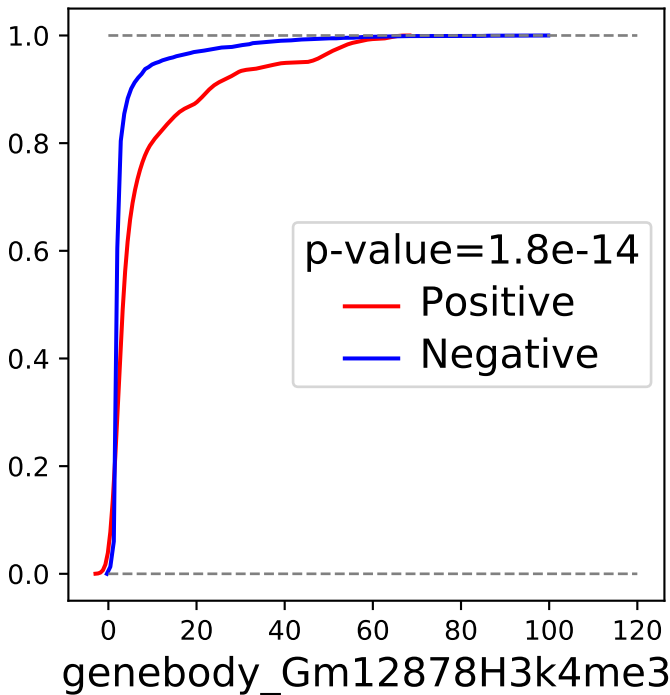

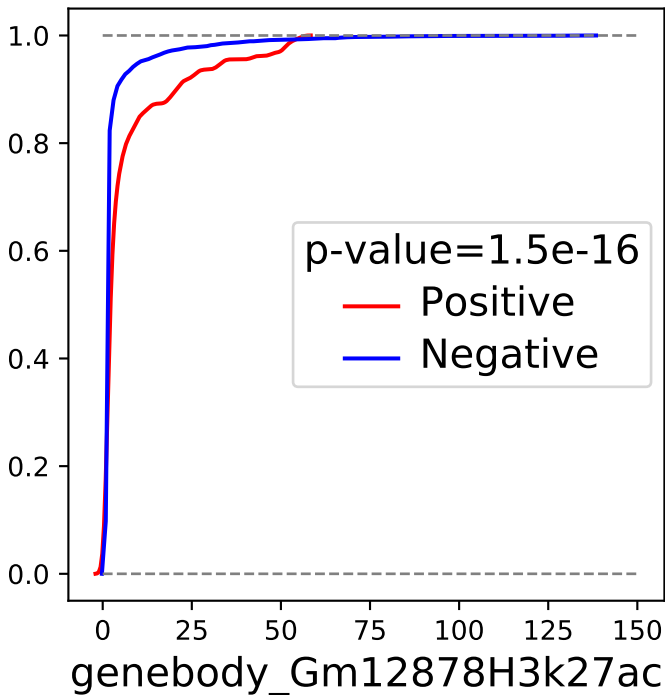

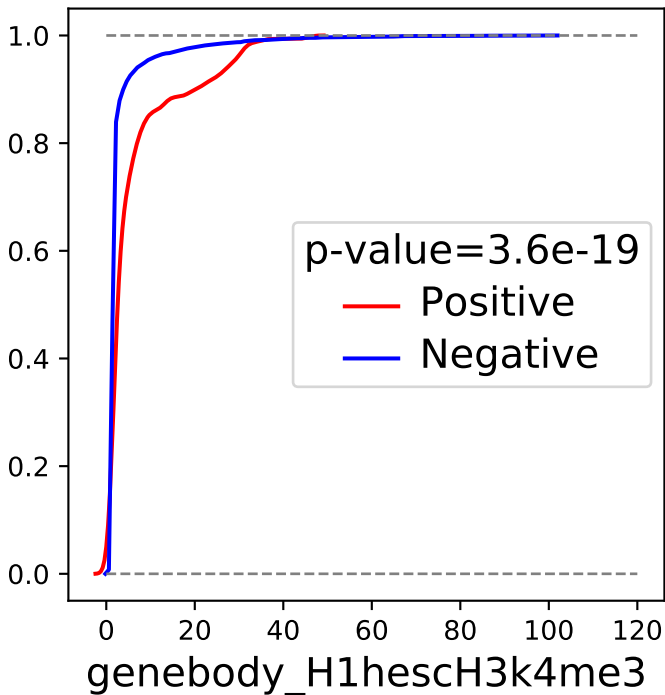

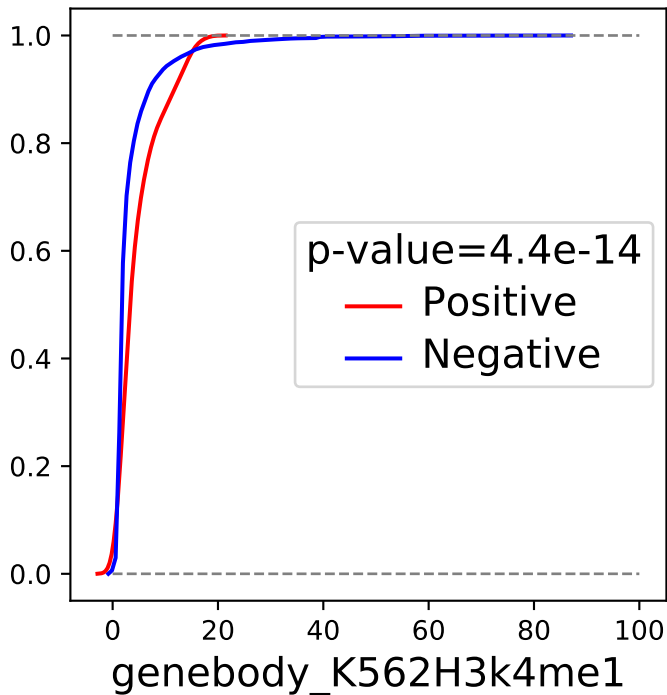

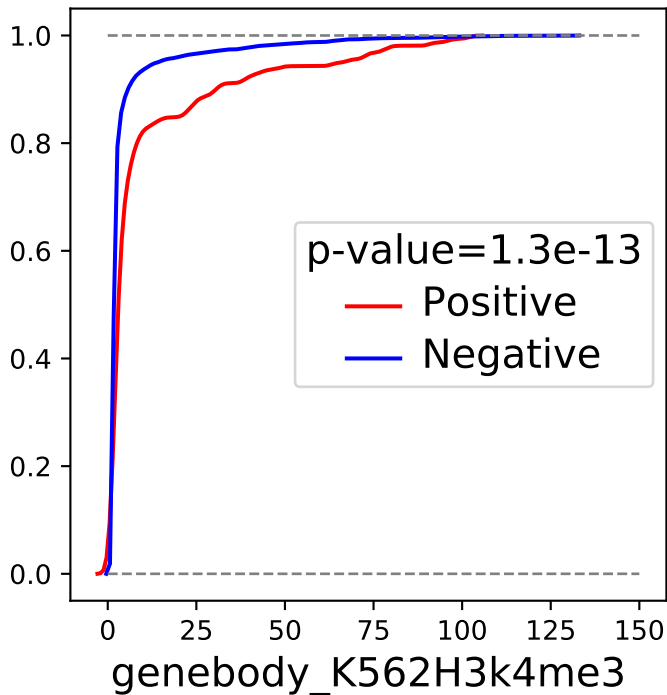

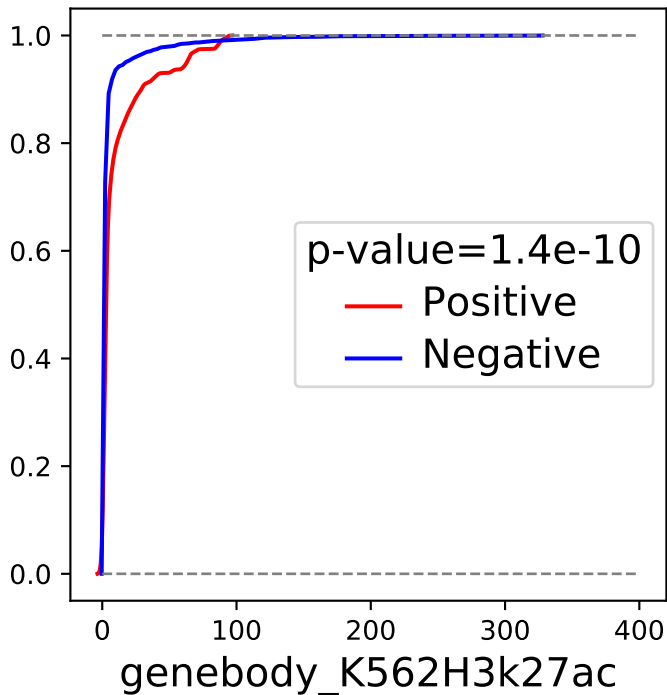

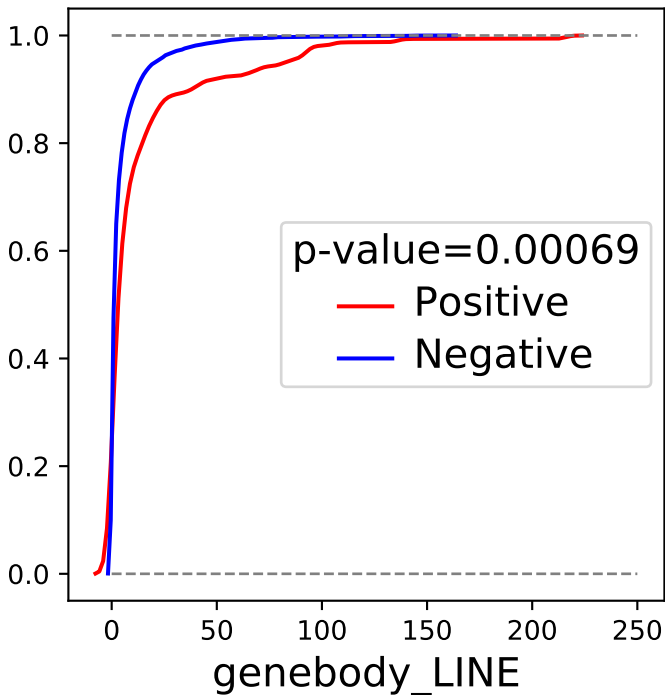

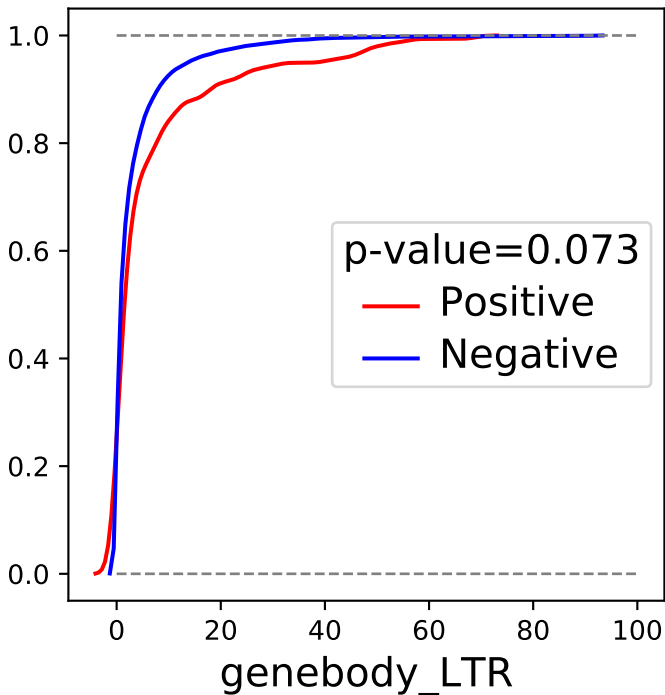

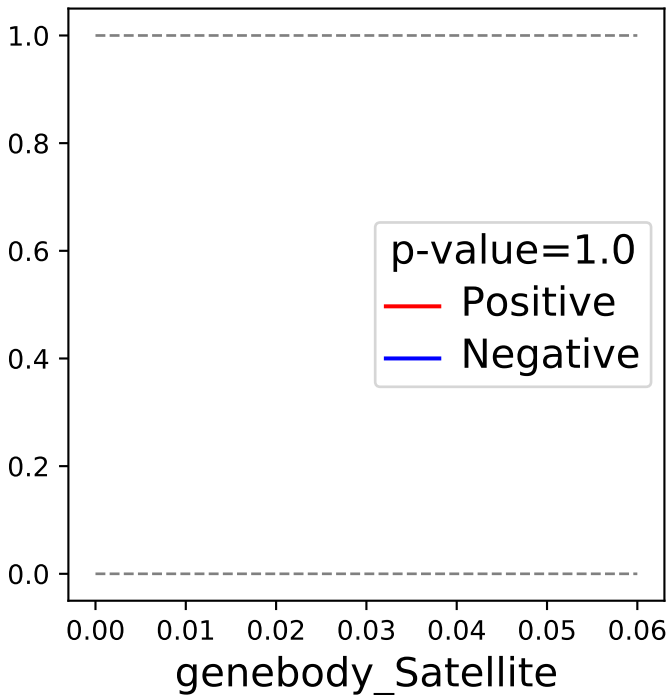

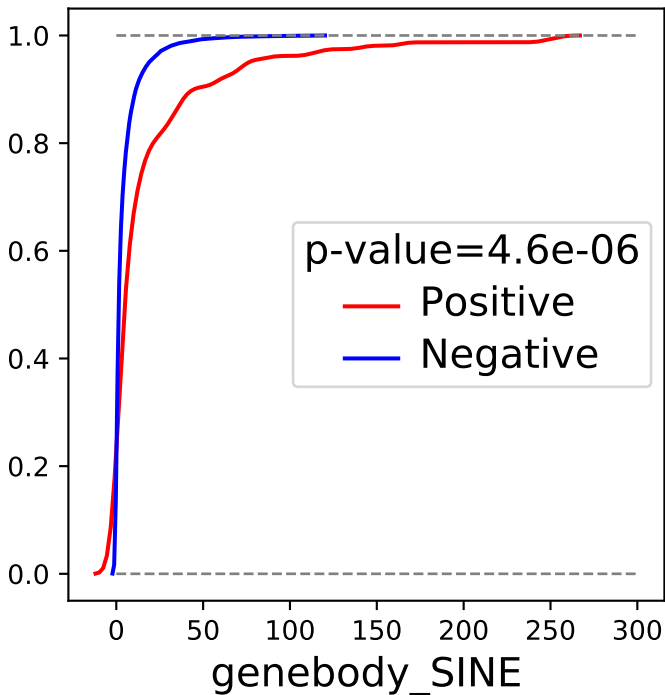

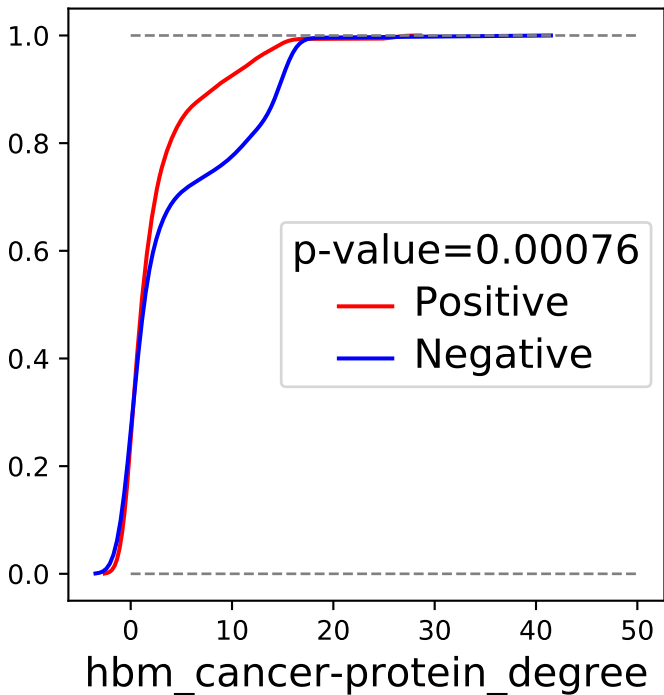

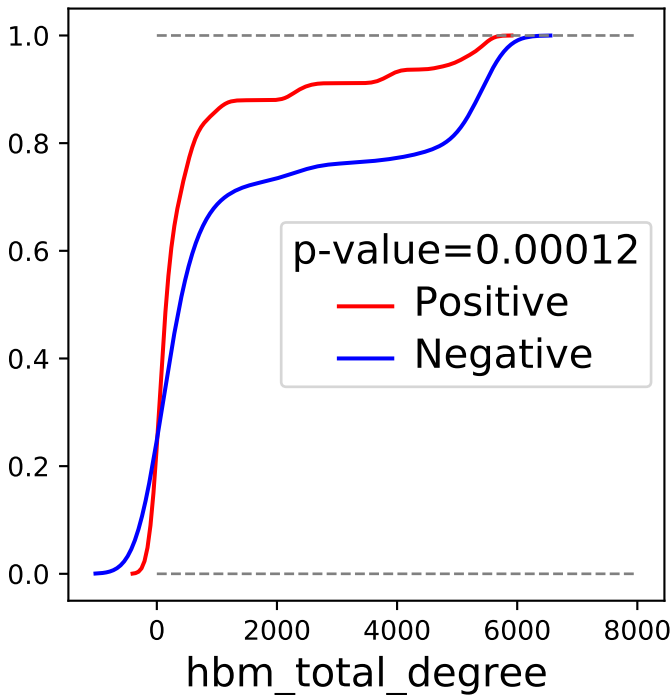

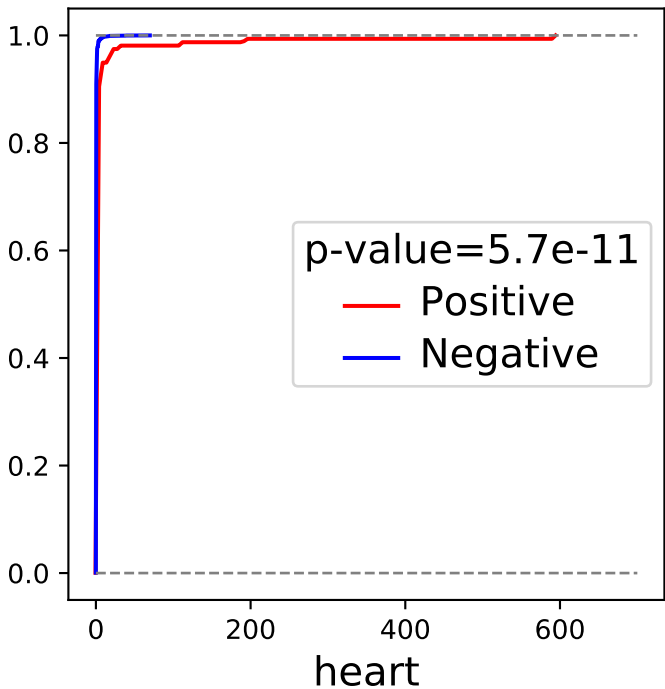

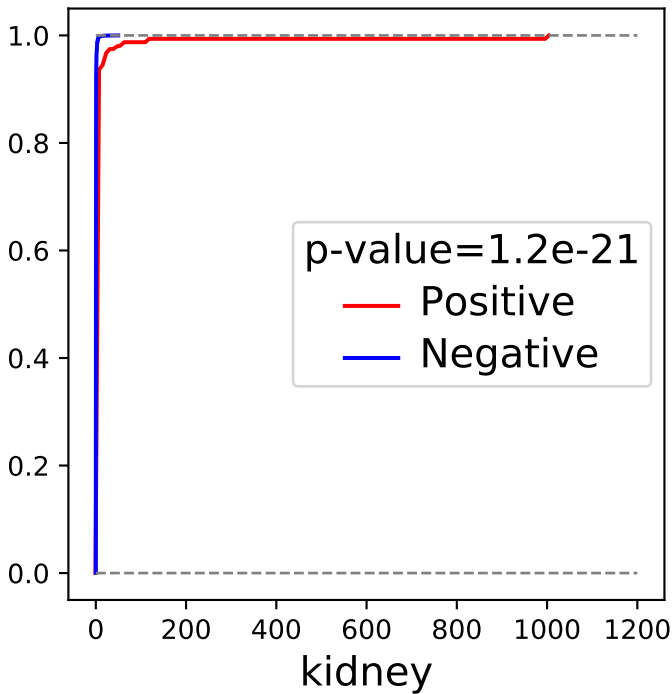

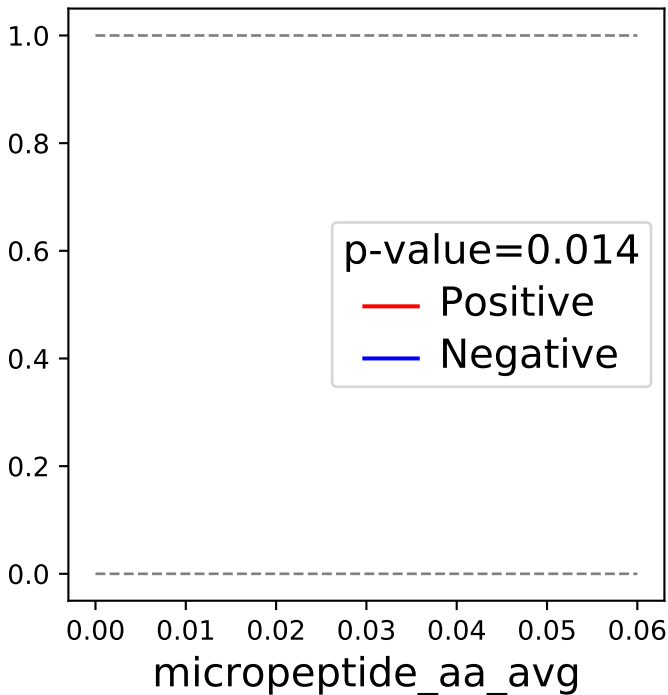

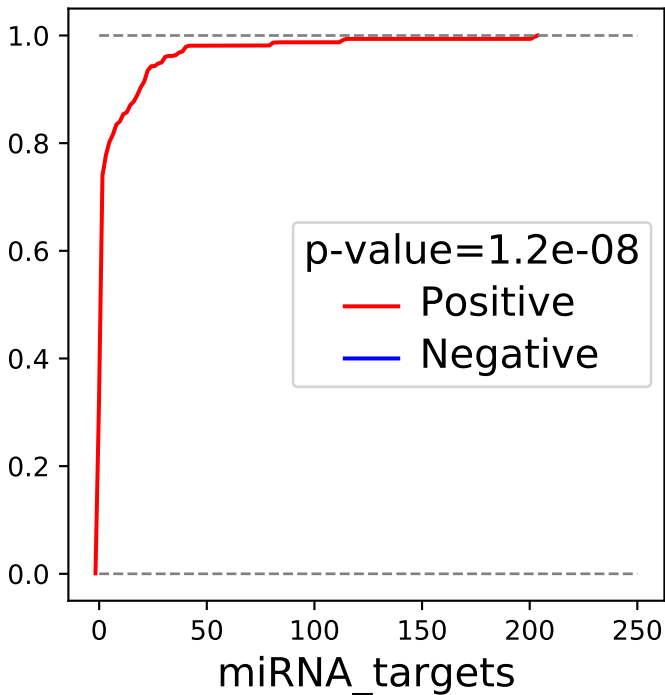

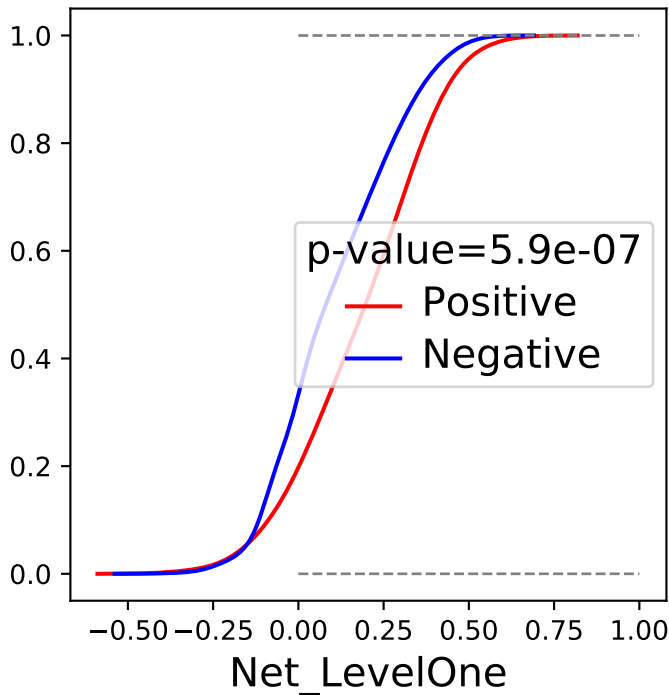

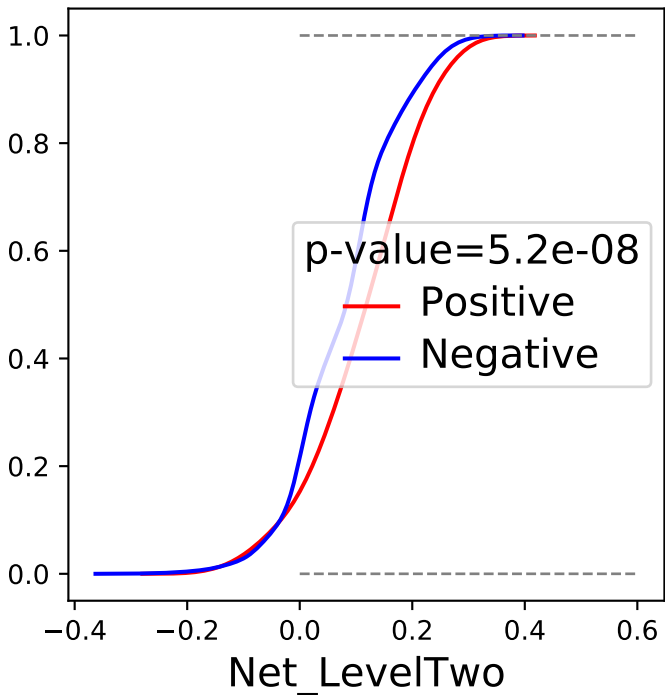

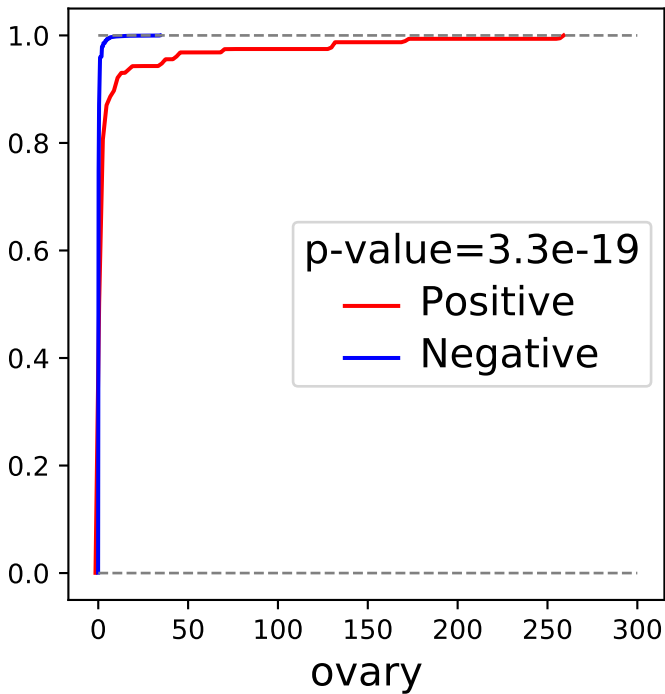

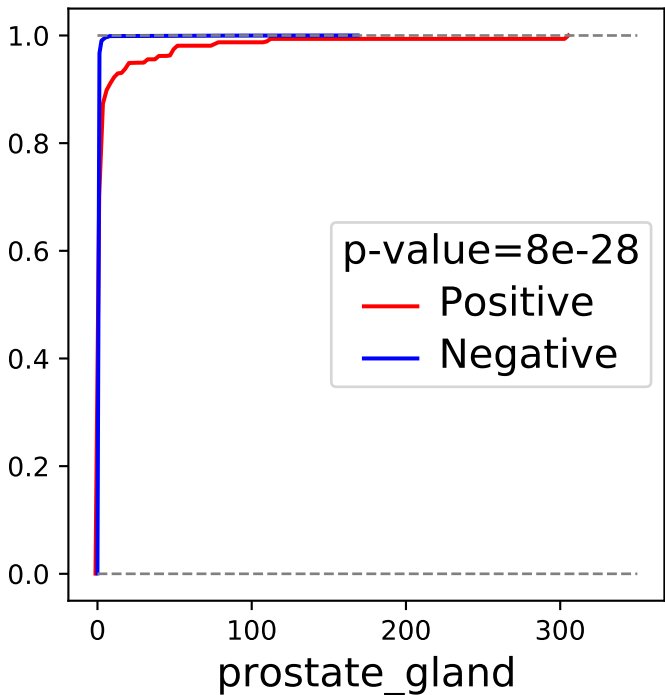

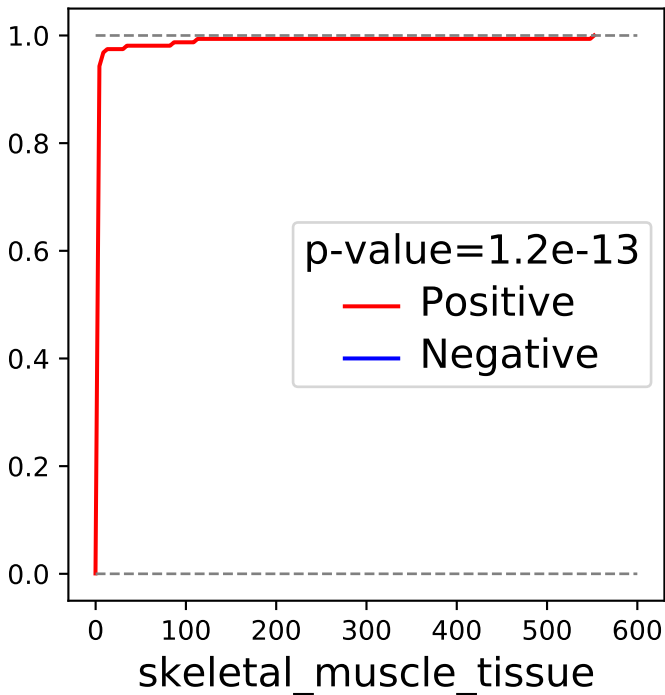

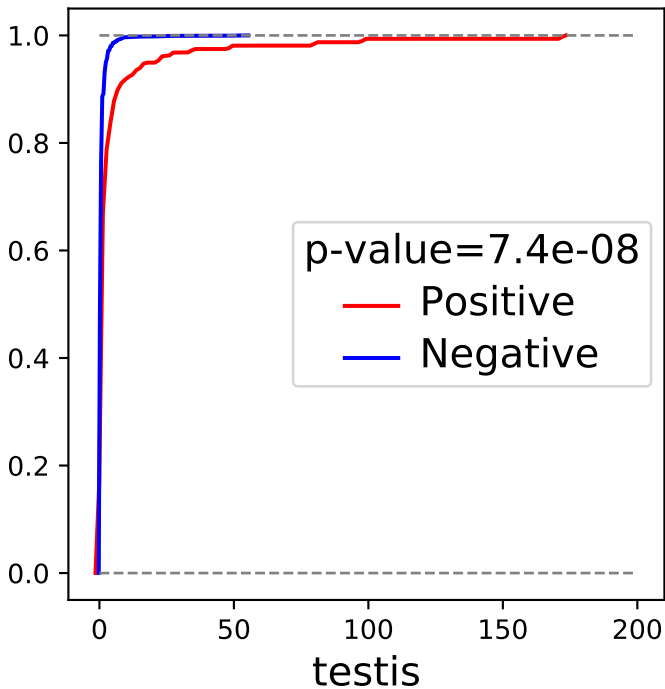

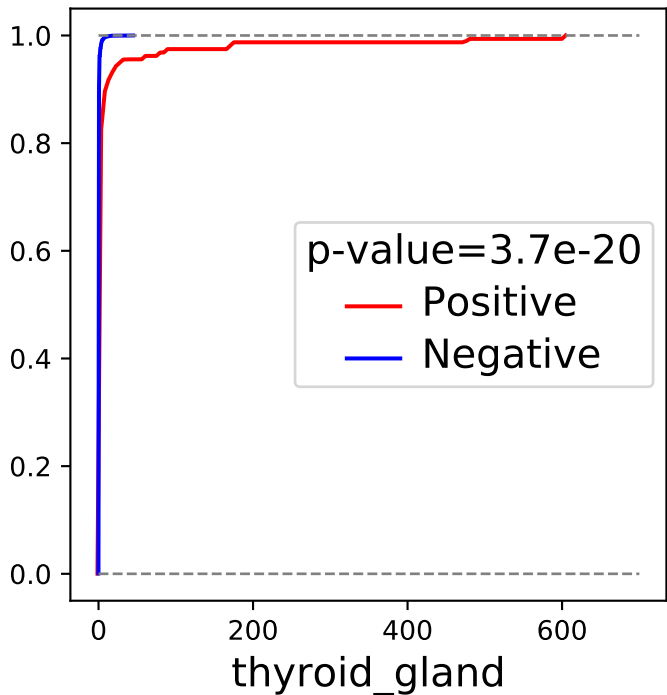

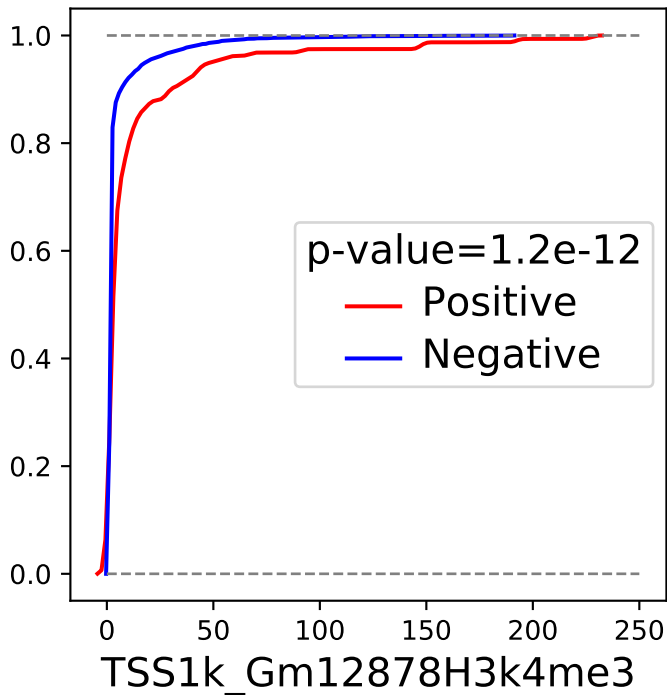

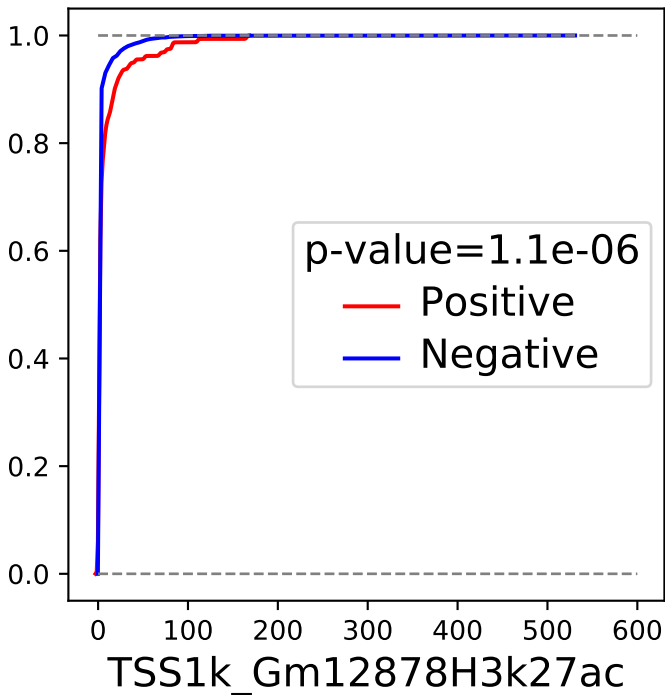

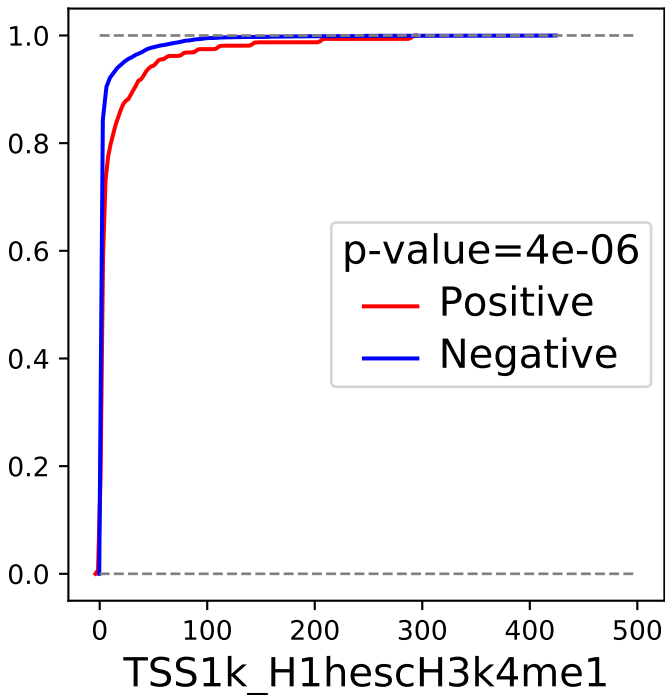

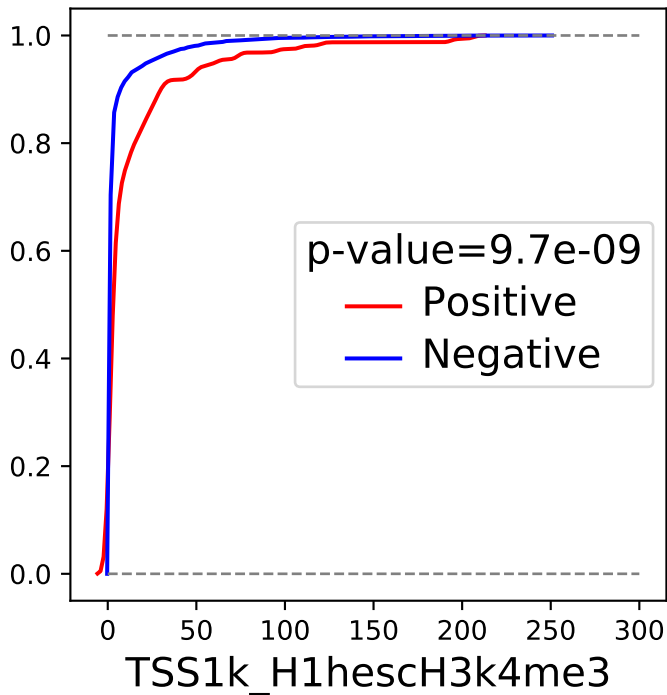

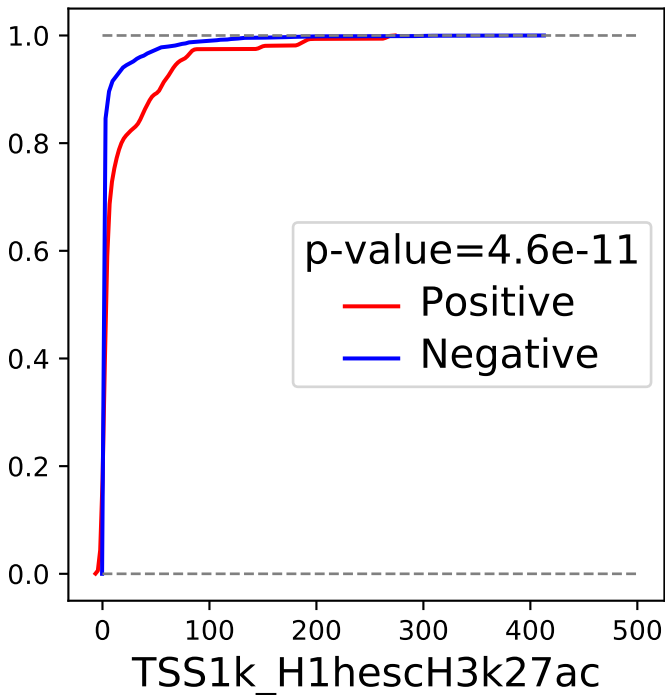

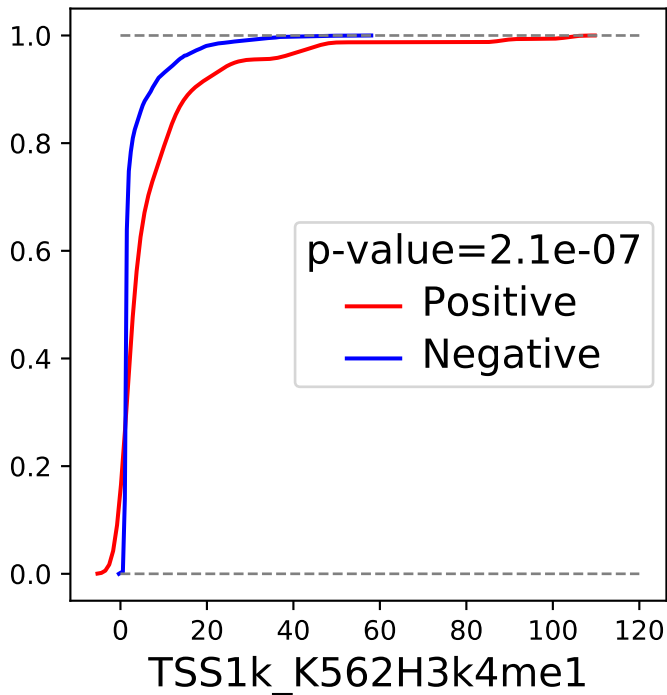

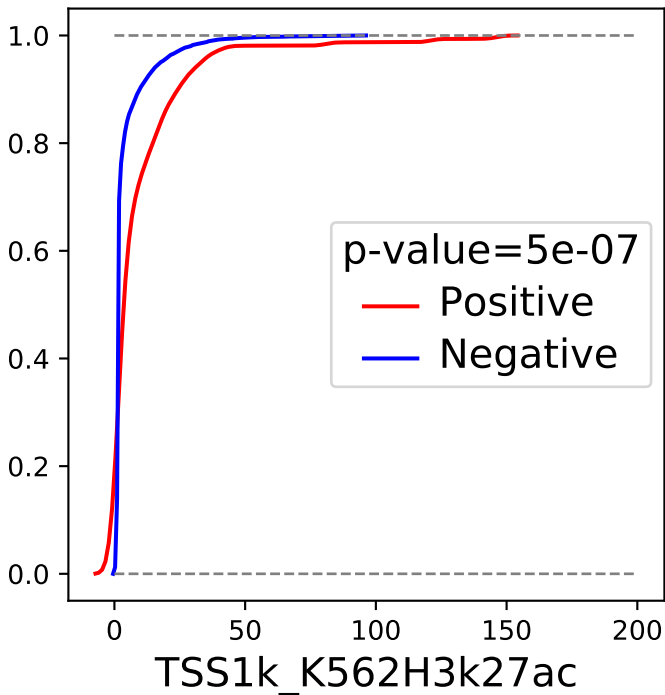

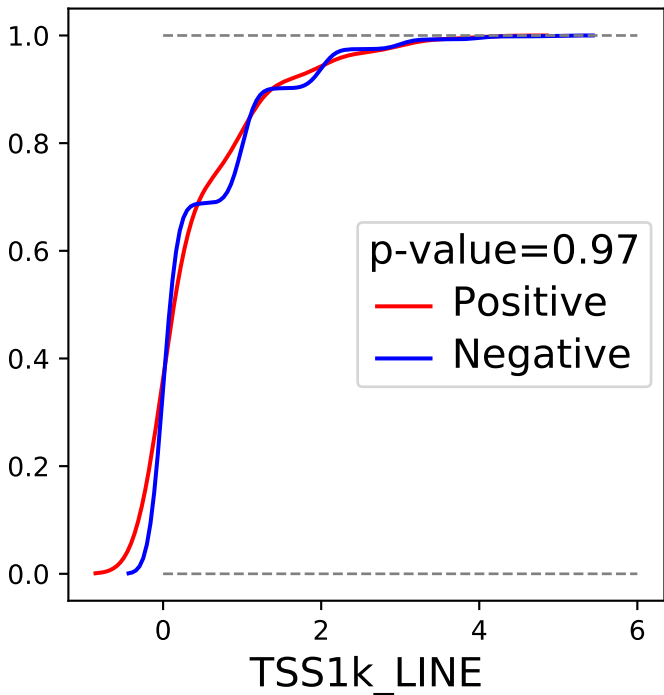

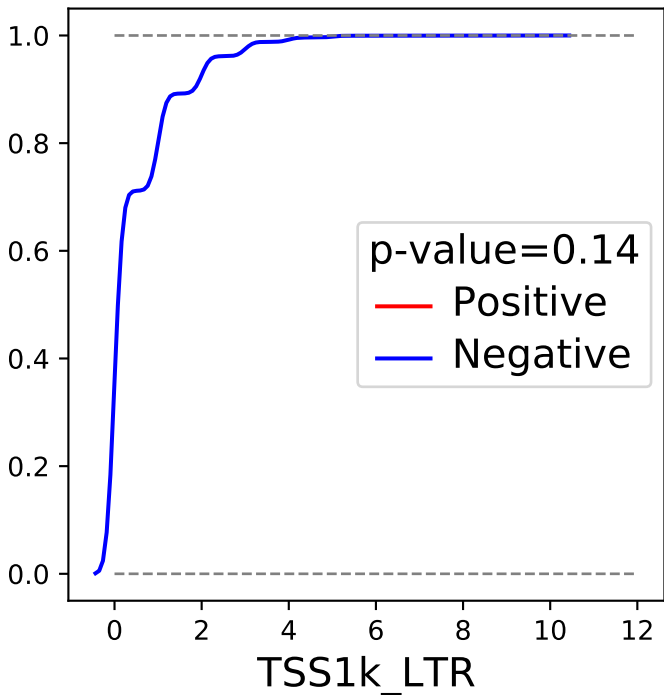

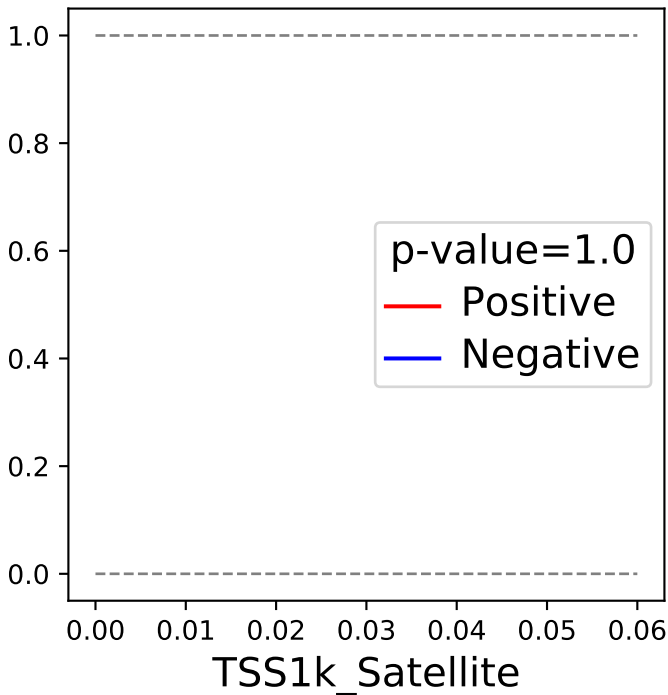

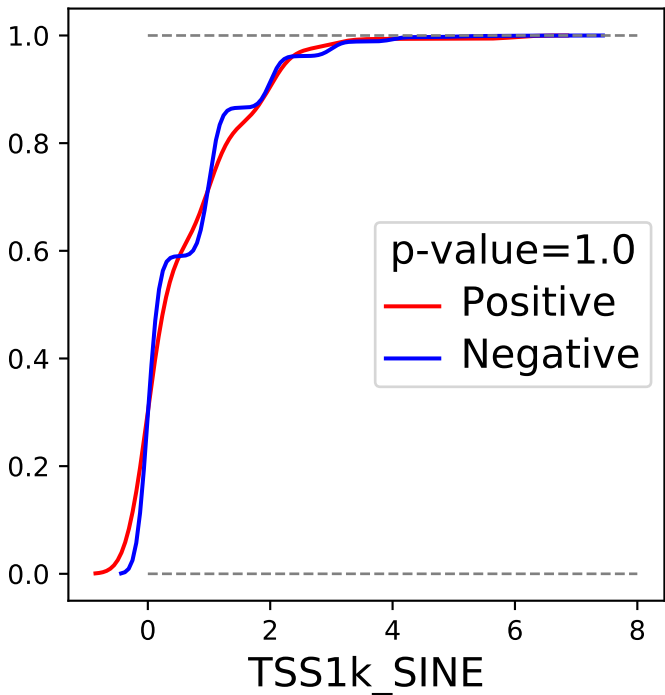

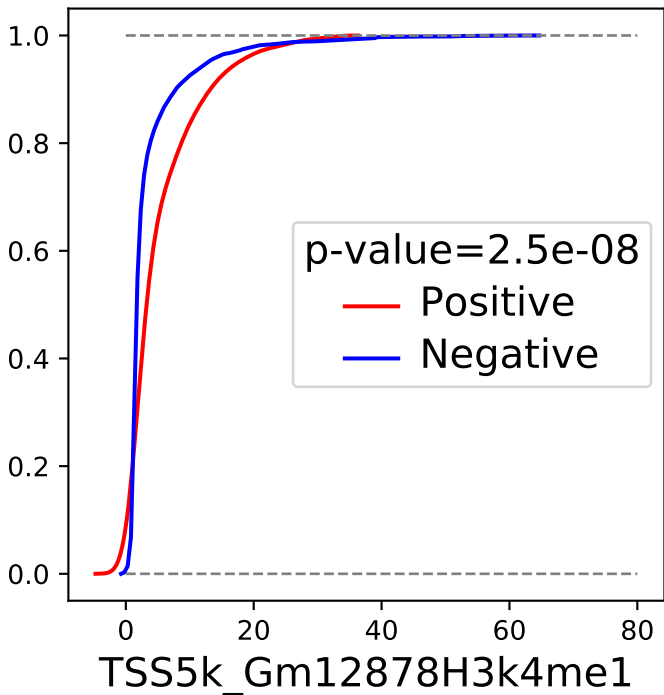

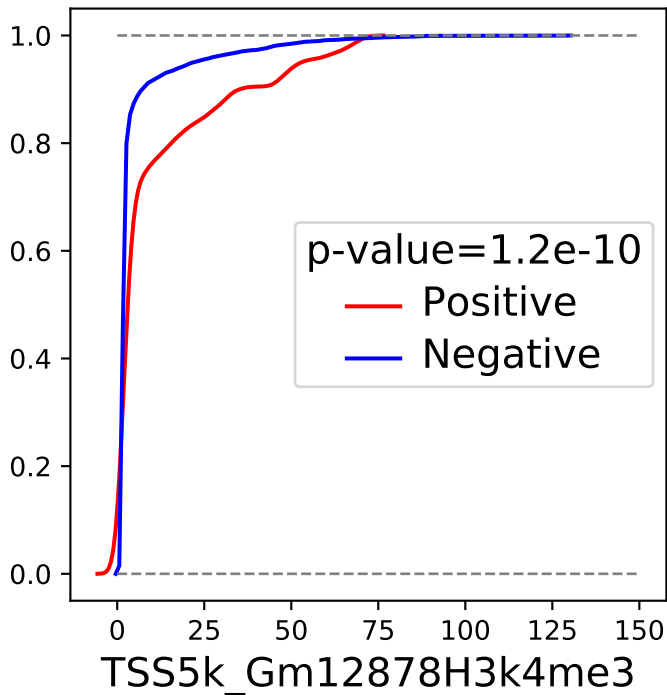

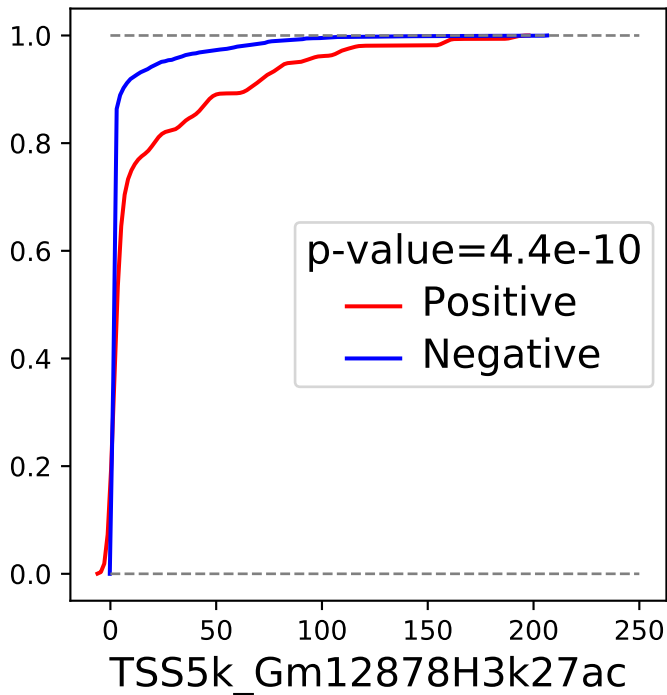

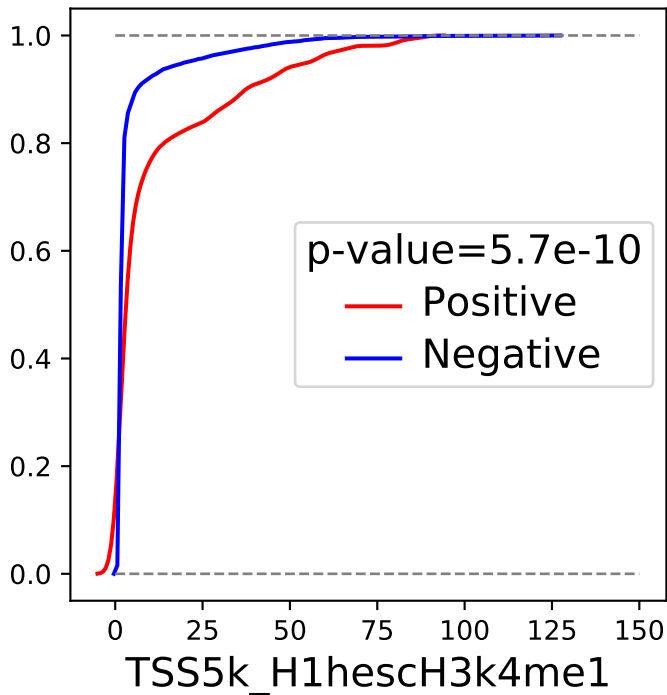

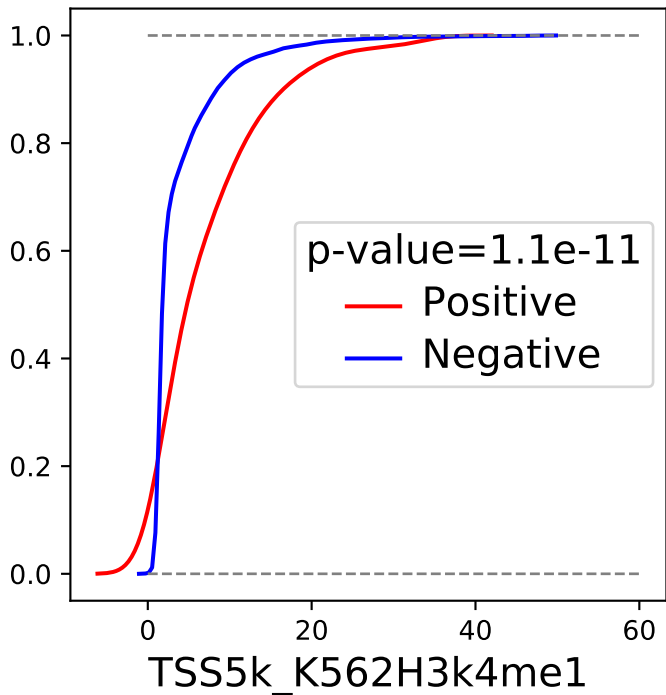

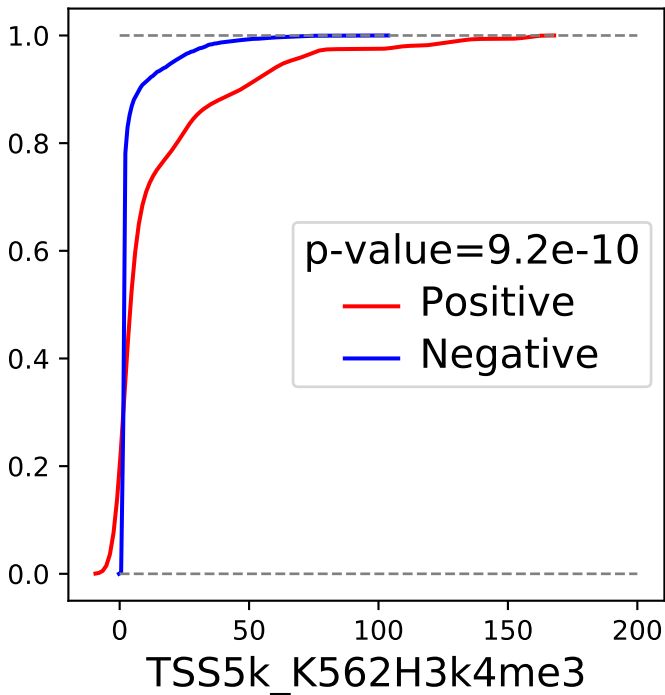

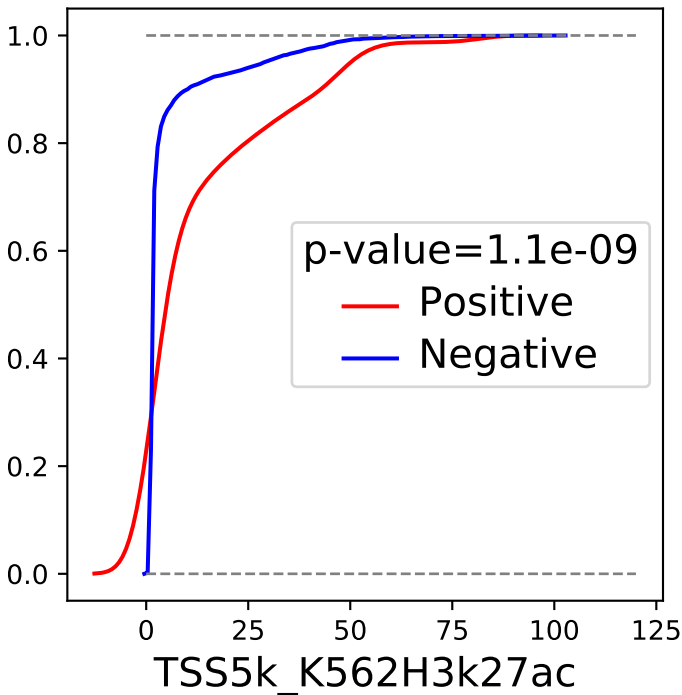

Supplement: Data Sheet 5 — Cumulative curves of positive and negative lncRNAs for all features. PDF 538KB [file DataSheet_5.pdf]

A

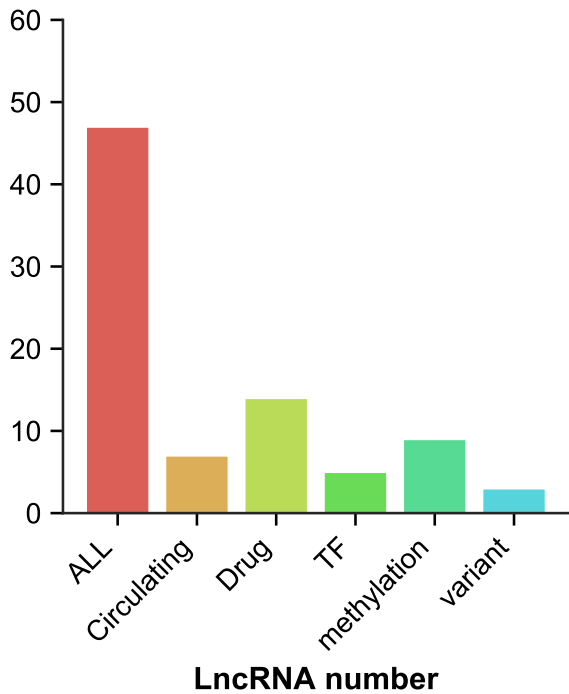

B

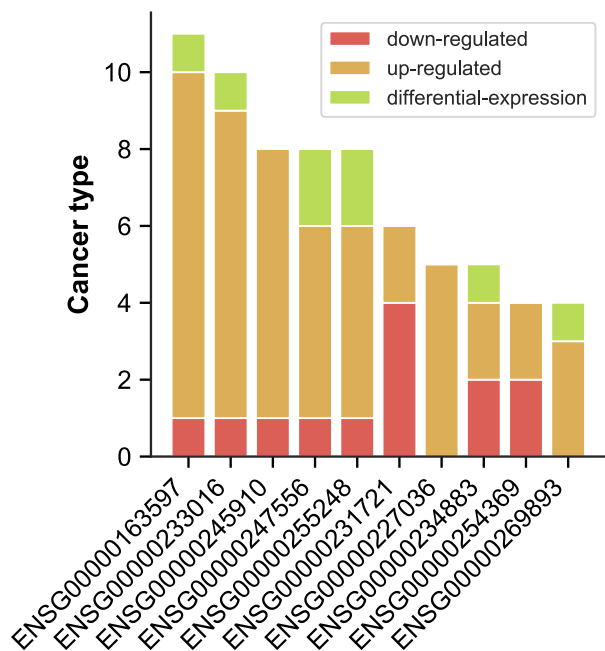

C

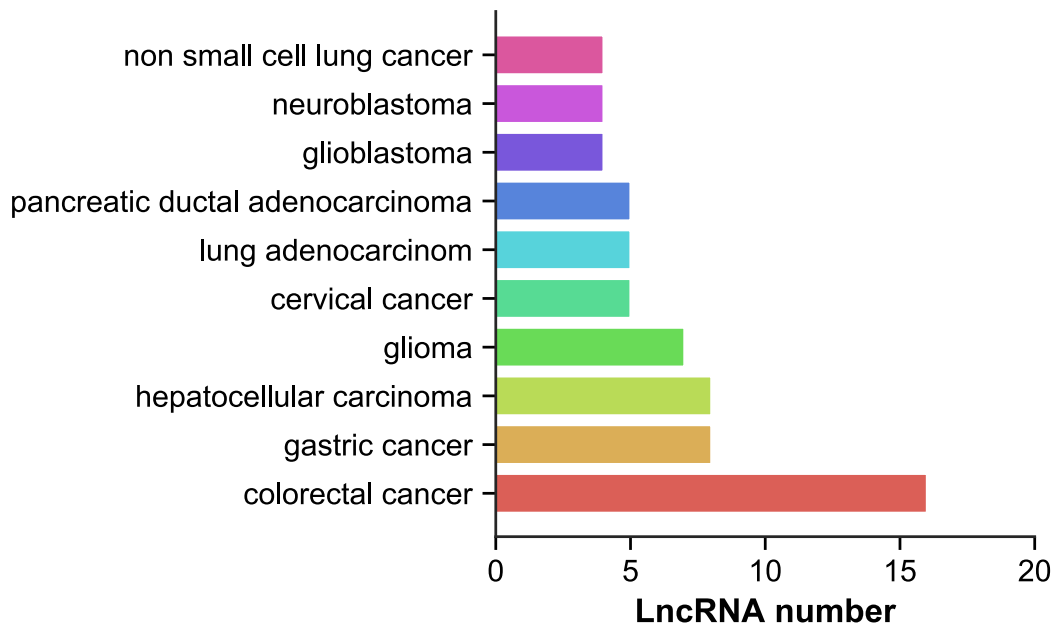

Supplement: Data Sheet 8 — Statistics of the interactions with Lnc2Cancer v2.0. PDF 207KB [file DataSheet_8.pdf]
